# Genome-Driven Discovery of Anti-MDR Bacterial Heptapeptides from a Cold-Seep-Derived *Bacillus* Strain

**DOI:** 10.3390/molecules31030547

**Published:** 2026-02-04

**Authors:** Hongcheng Li, Yongmeng Cheng, Kaishuai Xing, Wenli Li, Fei Xiao

**Affiliations:** 1 Key Laboratory of Marine Drugs, Ministry of Education of China, School of Medicine and Pharmacy, Ocean University of China, Qingdao 266003, China; lihongcheng@stu.ouc.edu.cn (H.L.); 18945124627@163.com (Y.C.); xingkaishuai566@126.com (K.X.); liwenli@ouc.edu.cn (W.L.); 2 Laboratory for Marine Drugs and Bioproducts, Qingdao Marine Science and Technology Center, Qingdao 266237, China; 3 State Key Laboratory for Crop Stress Resistance and High-Efficiency Production, College of Chemistry & Pharmacy, Northwest A&F University, Yangling 712100, China; 4 Shaanxi Key Laboratory of Natural Products & Chemical Biology, College of Chemistry & Pharmacy, Northwest A&F University, Yangling 712100, China

**Keywords:** genome mining, anti-MDR bacterial activity, cold-seep, *Bacillus subtilis*, heptapeptide, biosynthetic pathway

## Abstract

With the increasing emergence of multidrug-resistant (MDR) bacteria, there is an urgent need to discover new antibiotics. In this study, genome mining coupled with anti-bacterial assay guided the targeted isolation of two new heptapeptides nobilamide Q3 (**1**) and R3 (**2**). These compounds were identified as new stereoisomers of the known scaffold A-3302-B (**3**). The structures of these compounds were elucidated through a combination of MS, NMR spectroscopy and Marfey’s analysis. Anti-MDR bacterial assays showed that compounds **2** and **3** exhibited effective growth inhibition against the Gram-positive MDR bacterial strain *Staphylococcus aureus* CCARM 3090 with MIC values of 3.25–6.5 μg/mL. Notably, our study reveals stereochemistry-dependent differences in their antibacterial activities, providing new insights into the structure–activity relationship of this class of peptides. Finally, an analysis of the biosynthetic gene cluster responsible for their production was conducted. This study underscores the significance of exploring cold-seep environments as a reservoir for discovering new antibiotics and provides a structural starting point for the future optimization of antimicrobial peptides.

## 1. Introduction

In recent decades, the misuse and overuse of antibiotics in humans have led to the emergence and global spread of multidrug-resistant (MDR) pathogens. MDR bacteria have now become one of the top global threats to public health, contributing to the deaths of 4.95 million people in 2019 [1,2]. The limited pipeline of new antibiotics has further accentuated the disparity between increasing bacterial resistance and the urgent need for effective antimicrobial therapies. Consequently, there is a pressing requirement for the development of novel antibiotics.

Marine microorganisms, especially those in deep-sea cold-seep environments, thrive under extreme conditions including perpetual darkness, low temperatures, high hydrostatic pressure, and low oxygen levels [3]. These harsh habitats compel microbes to develop unique biochemical and physiological adaptations, often resulting in the production of structurally novel bioactive natural products [4,5]. Recent studies have uncovered diverse untapped biosynthetic gene clusters (BGC) and rare metabolites from deep-sea microbes, including a polyphosphorylated peptide exhibiting protease inhibitory activity, highlighting their significant potential for drug discovery and development [6].

Lipopeptide natural products are compounds featuring a fatty acid moiety covalently linked to the N-terminal of a nonribosomal peptide chain, which are typically produced by nonribosomal peptide synthetase (NRPS) pathway [7]. The fatty acid chain is introduced by a starter condensation (C) domain at the initiation of NRPS and plays a significant role in their biological activities [8]. This structural class of compounds has been isolated from multiple bacterial and fungal genera. Representative examples include calcium-dependent antibiotics (daptomycin and malacidin) [9,10], broad-spectrum antifungal compounds (sallidins, laxaphycins, and pneumocandins) [11,12,13], and surfactins, which rank among the most effective biosurfactants currently available [14]. Due to their significant antimicrobial activity, lipopeptides have attracted significant attention as promising candidates for the development of novel antibiotics and antifungal drugs.

During our ongoing research to discover bioactive natural products, we conducted genome mining on *B. subtilis* 4-L-22, isolated from a cold-seep environment. Detailed analysis revealed 4-L-22 harbors a nobilamide BGC (*nbl_BS_*) with the remarkable substrate promiscuity, indicating that this strain may generate new nobilamide analogs. Guided by this analysis, we probed the production of new nobilamide analogs in *B. subtilis* 4-L-22, leading to the identification of two new heptapeptides, nobilamide Q3 (**1**) and R3 (**2**), which are stereoisomers of the known compound A-3302-B (**3**) [15]. Herein, we report the isolation, structure elucidation, the anti-MDR bacterial activity, and the proposed biosynthetic pathway of **1**–**3**.

## 2. Results and Discussion

### 2.1. Identification and Fermentation of Strain 4-L-22

**Figure 1:**
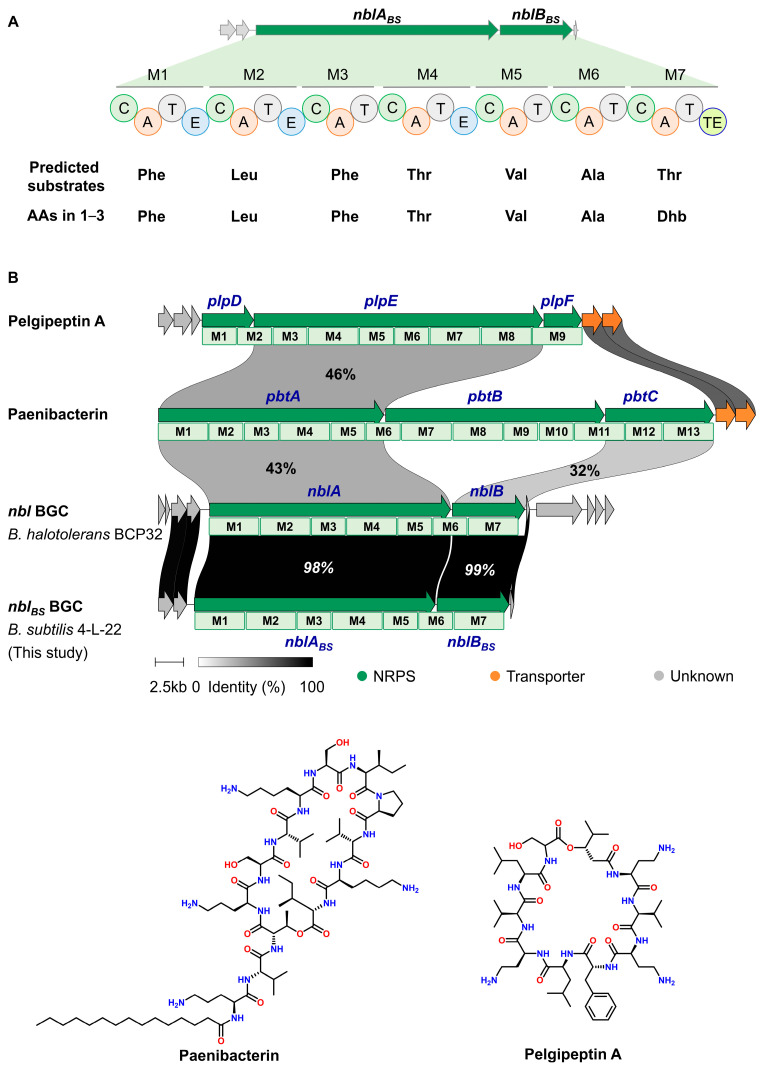
(**A**) The deduced biosynthetic gene cluster of nobilamides (**1**–**3**); (**B**) Comparative analysis of the *nbl_BS_* BGC with other homologous BGCs. Abbreviations: C, condensation domain; A, adenylation domain; T, peptidyl carrier protein; E, epimerization domain; TE, thioesterase domain.

The strain 4-L-22 was isolated from the cold-seep in the South China Sea. Preliminary bioassays of the crude fermentation extracts demonstrated that 4-L-22 has the potential to produce antimicrobial substances. Therefore, the genome of 4-L-22 was sequenced. The strain 4-L-22 was identified as *Bacillus subtilis* based on 16S rRNA gene sequence alignment using the EzTaxon-e server [16] (99.93% identity to that of *B. subtilis* NCIB 3610^T^, Table S1, Figure S1) and designated as *B. subtilis* 4-L-22. In silico analysis of the annotated draft genome allowed identification of an NRPS BGC (GenBank ID: PX436165-PX436169). This BGC was located within a 31-kb DNA region comprising five open reading frames (ORFs), including two NRPS genes (*nblA_BS_* and *nblB_BS_*, Figure 1A, Table S3). No obvious tailoring genes were found adjacent to *nblA_BS_* and *nblB_BS_*. The core NRPS enzymes, NblA_BS_ and NblB_BS_, share 98–99% sequence identity with those from the recently characterized nobilamide (*nbl*) BGC in *B. halotolerans* BCP32 (GenBank: PQ787232), confirming its identity [17]. In contrast, they exhibit a lower identity of 35–50% to the NRPS enzymes from the paenibacterin (MIBiG Accession: BGC0000400, *pbt* BGC from *Paenibacillus* sp. OSY-SE) [18] and pelgipeptin (MIBiG Accession: BGC0000403, *plp* BGC from *Paenibacillus elgii*) BGCs (Figure 1B) [19].

Sequence analysis revealed that *nblA_BS_* encodes a five-module NRPS with an *N*-terminal condensation (C) domain, which serves as the typical starting module for *N*-acylated nonribosomal peptides assembly [8]. The *nblB_BS_* gene encodes a two-module NRPS bearing a C-terminal thioesterase (TE) domain, which mediates product release and is typically found in PKS or NRPS. Substrate specificities of the A-domains in NblA_BS_ and NblB_BS_ were predicted using NRPSpredictor2 [20], suggesting their preferences for Phe^1^, Leu^2^, Phe^3^, Thr^4^, Val^5^, Ala^6^ and Thr^7^, respectively (Figure 1A). Recent reports of 18 and 84 nobilamide analogs from the cucumber epiphyte *Bacillus* sp. G2112 and the deep-sea strain *B. halotolerans* BCP32, respectively, underscore the remarkable substrate promiscuity of the *nbl* gene cluster [17,21]. This biosynthetic flexibility suggests that the *nbl_BS_* BGC identified in our study likely also directs the production of a wider array of nobilamide derivatives beyond those isolated. Motivated by this observation, we probed the production of new nobilamide analogs in *B. subtilis* 4-L-22.

**Figure 2:**
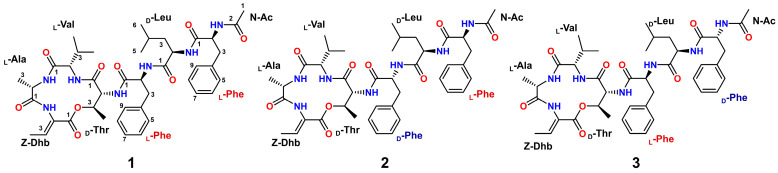
Structures of compounds **1**–**3**.

To mine the nobilamide analogs with anti-MDR activity produced by *B. subtilis* 4-L-22, we conducted fermentations in Landy medium collected at 6, 9, 12, 18, 24, and 36 h. The resulting fermentation products were subjected to antibacterial tests against *Staphylococcus aureus* CCARM 3090, *Enterococcus faecalis* CCARM 5172, *Klebsiella pneumoniae* ATCC 29213 and *Pseudomonas aeruginosa* 15690. All fermentation products exhibited significant anti-MDR activity, with the 24 h fermentation products showing the optimal activity (Figure S3). Meanwhile, the HPLC-HRESIMS profile revealed three peaks, among which the peak at *m*/*z* 804.4277 [M + H]^+^—with a calculated molecular formula of C_42_H_58_N_7_O_9_—might consist of seven amino acid residues and could be a product of the *nbl_BS_* BGC. Therefore, a large-scale fermentation (10 L) of *B. subtilis* 4-L-22 in Landy medium was carried out and collected after 24 h. The crude extracts were then subjected to reversed-phase open column chromatography followed by HPLC purification to afford compounds **1**–**3** (Figure 2).

### 2.2. Structure Elucidation

**Figure 3:**
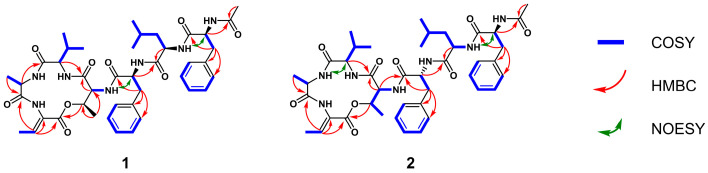
The key ^1^H-^1^H COSY, HMBC and NOESY correlations for compounds **1** and **2**.

Compound **1** was isolated as a white amorphous powder. The HRESIMS spectrum showed a protonated molecule [M + H]^+^ at *m*/*z* 804.4277, indicating the molecular formula of C_42_H_57_O_9_N_7_, which required 18 degrees of unsaturation. The ^1^H NMR spectra in DMSO-*d*_6_ (Table 1) showed seven exchangeable amide NH protons (*δ*_H_ 7.94, 1H, s; 8.36, 1H, d, *J* = 9.3 Hz; 8.41, 1H, br s; 8.56, 1H, br s; 8.86, 1H, s; 9.13, 1H, br s; 9.52, 1H, br s), and six *α*-amino protons (*δ*_H_ 3.78, 1H, d, *J* = 5.2 Hz; 3.91, 1H, m; 3.94, 1H, m; 4.26, 1H, m; 4.64, 1 H, m; 4.66, 1H, m). Careful analysis of the ^13^C and HSQC NMR data of compound **1** revealed forty-two carbon signals, including seven carbonyls (*δ*_C_ 164.5, 167.7, 170.8, 171.0, 171.0, 171.6, 172.0), fourteen aromatic carbons (*δ*_C_ 125.5, 126.0, 126.2, 127.4, 127.4, 128.0, 128.0, 129.2, 129.2, 130.3, 130.3, 134.4, 136.3, 138.2), nine methines (*δ*_C_ 23.2, 29.6, 50.5, 52.2, 52.6, 54.0, 58.5, 61.3, 72.0), three methylenes (*δ*_C_ 37.0, 38.0, 39.2), and eight methyls (*δ*_C_ 15.1, 16.6, 17.5, 19.0, 19.2, 22.0, 22.4, 23.3). Detailed analysis of the aforementioned data indicated that compound **1** is a heptapeptide comprising seven amino acid residues, confirming the presence of *N*-acetyl-phenylalanine (*N*-Ac-Phe), leucine (Leu), phenylalanine (Phe), threonine (Thr), valine (Val), alanine (Ala) and dehydrobutyrine (Dhb) (Figure 2). The connections of the Leu-Phe and Thr-Val-Ala-Dhb fragments were established by the key HMBC correlations from NH (*δ*_H_ 9.52) of Phe to C1 (*δ*_C_ 171.6) of Leu, H2 (*δ*_H_ 3.94) of Val to C1 (*δ*_C_ 167.2) of Thr, H2 (*δ*_H_ 4.26) of Ala to C1 (*δ*_C_ 170.6) of Val and H3 (*δ*_H_ 6.63) of Dhb to C1 (*δ*_C_ 170.3) of Ala. Subsequently, *N*-Ac-Phe was connected to the N-terminal of Leu to establish the *N*-Ac-Phe-Leu-Phe fragments by NOESY correlations from NH (*δ*_H_ 8.41) of Leu to H2 (*δ*_H_ 4.64) of *N*-Ac-Phe, and then this fragment was extended to *N*-Ac-Phe-Leu-Phe-Thr-Val-Ala-Dhb based on NOESY correlations from NH (*δ*_H_ 7.94) of Thr to H2 (*δ*_H_ 4.66) of Phe. Finally, the HMBC correlation from H3 (*δ*_H_ 3.75) of Thr to C1 (*δ*_C_ 164.0) of Dhb allowed the linear fragment to be closed through ester bond linkage between them (Figure 3). Furthermore, we conducted MS^2^ analysis of compound **1**, and the results confirmed the peptide sequence connectivity (Figure S12). The planar structure of compound **1** was identical to the known compound A-3302-B [15]. The ^1^H and ^13^C NMR data of **1** are given in Table 1.

To determine the absolute configurations of the amino acid residues in **1**, Marfey’s reaction was performed. The acid hydrolysate of **1** and standard amino acids were reacted with _L_-FDAA. HPLC analysis of the reaction mixtures led to the identification of _L_-Ala, _L_-Val, _D_-Thr, _L_-Phe and _D_-Leu (Figure S29), indicating that both phenylalanine residues are L-configured. In contrast, the phenylalanine residues in A-3302-B were assigned as L- and D-configured, respectively [15]. Thus, compound **1** was identified as a new stereoisomer of the known compound A-3302-B (**3**), *N*-Ac-_L_-Phe-_D_-Leu-_L_-Phe-_D_-Thr-_L_-Val-_L_-Ala-Dhb, and named nobilamide Q3.

Compounds **2** and **3** were both obtained as white amorphous powders. Their molecular weights are identical to that of compound **1** (Figures S14 and S22), indicating a molecular formula of C_42_H_57_O_9_N_7_ with 18 degrees of unsaturation. Combined analysis of 1D and 2D NMR data of compounds **2** and **3** indicated that they shared the same planar structure as **1**. We then used Marfey’s method to determine the absolute configurations of **2** and **3**. HPLC analysis of the reaction mixtures and standard derivatives revealed the presence of both _L_-Phe and _D_-Phe in **2** and **3**, in contrast to compound **1**, which contains only _L_-Phe. Thus, **2** and **3** are configurational isomers of **1**, differing in the stereochemistry of their Phe residues. Further comparison of the ^1^H NMR data with those reported in the literature confirmed compound **3** was identical to the known heptapeptide A-3302-B/TL-119 [22,23]. Consequently, compound **2** was established as a new stereoisomer of A-3302-B (**3**), *N*-Ac-_L_-Phe-_D_-Leu-_D_-Phe-_D_-Thr-_L_-Val-_L_-Ala-Dhb, and designated as nobilamide R3.

The stereochemical variations among isomers **1**–**3** induced noticeable differences in their NMR spectra (Table 1). A comparison of the ^1^H NMR data for the new stereoisomers, nobilamide Q3 (**1**) and R3 (**2**), reveals the diagnostic impact of the Phe^3^ configuration. Specifically, the *α*-proton (H-2) of the _D_-Phe^3^ residue in nobilamide R3 (**2**) at *δ*_H_ 4.40 ppm is significantly shifted upfield compared to the _L_-Phe^3^ in nobilamide Q3 (**1**) at *δ*_H_ 4.66 ppm. Similarly, the amide proton of _D_-Phe^3^ in **2** (*δ*_H_ 9.32 ppm) is downfield compared to that in **1** (*δ*_H_ 9.52 ppm). This upfield shift is characteristic of a D-amino acid residue within a peptide macrocycle, which alters the local conformation and places the *α*-proton in a different magnetic environment. This observation further supports our stereochemical assignment based on Marfey’s analysis.

### 2.3. The Proposed Biosynthetic Pathway of ***1**–**3***

**Figure 4:**
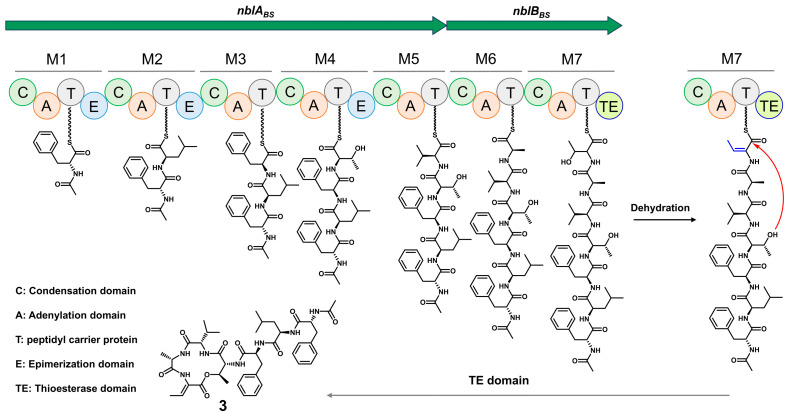
The proposed biosynthetic pathway of A-3302-B (**3**). The red arrow indicates the intramolecular nucleophilic attack from the side-chain hydroxyl group of Thr^4^ onto the C-terminal thioester, which leads to macrocyclization via ester bond formation, catalyzed by the thioesterase (TE) domain.

Based on bioinformatic analysis, we propose a biosynthetic pathway for **1**–**3** (Figure 4). Biosynthesis is initiated by the starter condensation domain, which incorporates an acetyl group onto the Phe^1^ residue. In accordance with the co-linearity rule, six additional amino acids, including Leu^2^, Phe^3^, Thr^4^, Val^5^, Ala^6^, Thr^7^, are sequentially assembled to form a linear peptide intermediate. The Thr^7^ residue is then proposed to undergo dehydration to generate the Dhb^7^ residue [24]. Finally, macrocyclization via ester bond formation between Dhb^7^ and Thr^4^, catalyzed by a TE domain during product offloading, affords product **1**. The biosynthesis of the major product, A-3302-B (**3**) (*N*-Ac-_D_-Phe-_D_-Leu-_L_-Phe-_D_-Thr-_L_-Val-_L_-Ala-Dhb), is consistent with the predicted functions of the E domains in modules 1 and 2, and the absence of an E domain in module 3. The formation of its stereoisomers, nobilamide Q3 (**1**) and nobilamide R3 (**2**), likely arises from the imperfect fidelity of these domains. The failure of the E domain in module 1 could lead to the incorporation of _L_-Phe^1^, resulting in compound **1**. The generation of the _D_-Phe residue at position 3 in nobilamide R3 (**2**) is particularly intriguing due to the absence of a canonical epimerization (E) domain in Module 3. This discrepancy might be attributed to the substrate promiscuity of the adenylation (A) domain, which could directly select and activate _D_-Phe from the intracellular pool. Alternatively, it may involve a discrete, free-standing racemase encoded elsewhere in the genome that acts in trans, a phenomenon that has been observed in other *Bacillus* peptide biosynthetic systems [25].

### 2.4. Biological Activity of ***1**–**3***

As shown in Table 2, nobilamide R3 (**2**) and A-3302-B (**3**) exhibited potent antibacterial activity against the Gram-positive MDR strain *S. aureus* CCARM 3090, with MIC values of 3.25 and 6.25 µg/mL, respectively. These activities were comparable or superior to the positive control tetracycline (MIC = 6.25 µg/mL). In contrast, compound Q3 (**1**) showed only moderate activity against this strain (MIC = 25 µg/mL). None of the tested compounds showed significant activity against the Gram-negative strains (MIC > 25 µg/mL), consistent with the previous reports [15,17,21,26]. Structurally, nobilamide Q3 (**1**) and R3 (**2**) are stereoisomers of the known peptide A-3302-B (**3**), distinguished by the configurations of the Phe^1^ and Phe^3^ residues. A comparative analysis with A-3302-B (**3**) and other analogs highlights the critical role of stereochemistry on the bioactivity [27]. While **1**–**3** share the same planar structure, the specific D-configuration of Phe^3^ in R3 (**2**) appears to correlate with enhanced potency compared to the _L_-Phe^3^ in Q3 (**1**). This finding adds new knowledge regarding the stereochemical requirements for the antibacterial efficacy of this scaffold, which was not fully elucidated in previous studies. However, these compounds only had inhibitions against *S. aureus* CCARM, exhibiting a narrow antibacterial spectrum. This structure–activity relationship provides a valuable starting point for future mechanistic investigations and the semi-synthetic optimization of this peptide scaffold to develop more potent antimicrobial agents.

## 3. Materials and Methods

### 3.1. General Experimental Procedures

High-performance liquid chromatography (HPLC) was performed on an Agilent 1260 system (Agilent Technologies, Santa Clara, CA, USA) using a YMC-Triart C18 column (150 × 4.6 mm, 5 µm, 12 nm) at a flow rate of 1 mL/min. The semi-preparative HPLC separation was conducted on Hitachi Primaide system (Hitachi, Ltd., Tokyo, Japan) equipped with a preparative YMC-Pack ODS-A C18 column (250 × 4.6 mm, 5 µm, 12 nm) at a flow rate of 2 mL/min. 1D and 2D NMR were recorded in DMSO-*d*_6_ on a Bruker Avance III 600 NMR spectrometer (600 MHz for ^1^H and 150 MHz for ^13^C, Bruker Corporation, Billerica, MA, USA). HPLC-HRESIMS data were acquired using an Agilent 1290 UPLC system (Agilent Technologies, Santa Clara, CA, USA) coupled with a Q-TOF Ultima Global GAA076 LC-MS spectrometer. Octadecyl silyl (ODS) silica gel (40 μm, 120 Å, Ji Nan Bo Na Biological Technology, Jinan, China) was used for open column chromatography. The optical density (OD) was recorded on a BioTek Epoch 2 Microplate Spectrophotometer (Agilent Technologies, Santa Clara, CA, USA). The MDR bacterial strains, including *S. aureus* CCARM 3090, *E. coli* CCARM 1009, *E. faecalis* CCARM 5172, *E. faecium* CCARM 5203, *S. typhimurium* CCARM 8250, *K. pneumoniae* ATCC 13883, *M. luteus* ML01 and *P. aeruginosa* 15690 were obtained from Seoul Women’s University, Republic of Korea.

### 3.2. Bioinformatic Analysis

The whole genome sequence of *B. subtilis* 4-L-22 was analyzed using the online antiSMASH 7.0 (https://antismash.secondarymetabolites.org/#!/start (accessed on 15 September 2024)) to predict the biosynthetic landscape. The substrate specificity of NRPS was predicted using the NRPSpredictor2 tool (https://github.com/roettig/NRPSpredictor2, accessed on 17 September 2024).

### 3.3. Anti-MDR Bacterial Activity

To test the anti-MDR bacterial activity, the marine-derived bacteria were cultured in Landy medium for 3 days at 37 °C. The culture was extracted with ethyl acetate and concentrated using rotary evaporator at 35 °C. Then, the resulting crude extracts were diluted with methanol to 10 mg/mL. The MDR bacterial strains, including *S. aureus* CCARM 3090, *E. coli* CCARM 1009, *E. faecalis* CCARM 5172, *E. faecium* CCARM 5203, *S. typhimurium* CCARM 8250, *K. pneumoniae* ATCC 13883, *M. luteus* ML01 and *P. aeruginosa* 15690, were cultured overnight in LB medium at 37 °C. Subsequently, the tested strain was diluted with LB broth to 10^6^ CFU/mL. Each bacterial solution (10^6^ CFU/mL) was mixed with LB agar at a 1:1000 volume ratio to prepare the test plates. Finally, 10 μL of each sample was added to the sterilized filter paper (5 mm in diameter) placed on the plate and incubated at 37 °C. After 24 h, the diameters of the inhibition zone were measured.

To determine the Minimum Inhibitory Concentration (MIC) values of compounds **1**–**3** against MDR bacteria, the compounds were redissolved in DMSO and added to 96-well plates to achieve final concentration (25 μg/mL, 12.5 μg/mL, 6.25 μg/mL, 3.125 μg/mL, 1.5625 μg/mL, 0.78125 μg/mL), with a final volume of 100 μL per well. The plates were incubated at 37 °C overnight, and the absorbance at 620 nm was measured using a BioTek Epoch 2 Microplate Spectrophotometer. The MIC values were recorded as the lowest concentrations of the compounds that inhibit the growth of tested bacteria.

The positive control used for the tested strains were tetracycline at 3 mg/mL. Methanol and LB broth were used as negative and blank controls, respectively.

### 3.4. Fermentation, Fraction and Isolation

To optimize the fermentation time, *B. subtilis* 4-L-22 was fermented in Landy medium for 6, 9, 12, 18, 24, and 36 h, respectively. The anti-MDR bacterial activity of the crude extracts at different fermentation times was evaluated as described in Section 3.3.

For the isolation of bioactive compounds, *B. subtilis* 4-L-22 was fermented in Landy medium for 24 h at 37 °C. Then, the fermentation was extracted three times with 10 L ethyl acetate and concentrated by rotary evaporator. The crude extract was further partitioned between MeOH: H_2_O (90:10, *v*/*v*) and *n*-hexane to obtain two residues. The aqueous MeOH layer was subjected to a reversed-phase column chromatography and stepwise eluted with MeOH: H_2_O (20:80 to 100:0, *v*/*v*) to afford fourteen fractions (Fr. 1–14). Then the fourteen fractions were evaluated the anti-MDR bacterial activity as described in Section 3.3. Fraction 6 (Fr. 6), eluted with MeOH: H_2_O (65:35, *v*/*v*), was further purified via semi-preparative HPLC (ACN: H_2_O, 45:55, *v*/*v*, 1.5 mL/min) to yield **1** (5.8 mg), **2** (3.5 mg), **3** (3.3 mg), with purities > 98% based on HPLC analysis.

Nobilamide Q3 (**1**): white powder; UV (MeOH) λ_max_ (log ε) 215 (3.40), 265 (2.38) nm; ^1^H and ^13^C NMR data, Table 1; HRESIMS *m*/*z* 804.4277 [M + H]^+^ (calcd for C_42_H_57_O_9_N_7_ 804.4273).

Nobilamide R3 (**2**): white powder; UV (MeOH) λ_max_ (log ε) 215 (4.05), 260 (3.30) nm; ^1^H and ^13^C NMR data, Table 1; HRESIMS *m*/*z* 804.4277 [M + H]^+^ (calcd for C_42_H_57_O_9_N_7_ 804.4273).

A-3302-B (**3**): white powder; UV (MeOH) λ_max_ (log ε) 210 (3.91), 260 (2.81) nm; ^1^H and ^13^C NMR data, Table 1; HRESIMS *m*/*z* 804.4277 [M + H]^+^ (calcd for C_42_H_57_O_9_N_7_ 804.4273).

### 3.5. Marfey Analysis Method

Acid hydrolysis and Marfey’s reaction were carried out to determine the absolute configuration as previously reported [28]. Compounds **1**–**3** (200 μg of each compound) were dissolved in 1 mL of 6N HCl and hydrolyzed for 24 h at 100 °C. Then the HCl was removed by freeze-drying, respectively. The hydrolysis product was suspended in 100 μL H_2_O and supplemented with 50 μL of 1M NaHCO_3_. The mixture was reacted with 200 μL 1-fluoro-2,4 dinitrophenyl-5-_L_-alanine amide (_L_-FDAA, 1 mg/mL at acetone) and incubated at 50 °C for 60 min. Subsequently, the reaction was quenched with 25 μL of 2N HCl. The crude mixture was freeze-dried to dryness, and dissolved in MeOH for further analysis. Amino acid standards were treated with _L_-FDAA as described above, yielding _L_-FDAA derivatives. Marfey’s derivatives of hydrolysate and standards were subjected to HPLC analysis eluted with ACN: H_2_O (30:70 to 100:0, *v*/*v*, supplemented with 0.05% trifluoroacetic acid) at 1 mL/min.

## 4. Conclusions

In summary, by genome mining coupled with the anti-MDR bacterial activities, chemical investigation of cold-seep-derived *B. subtilis* 4-L-22 led to the identification of two new heptapeptides, nobilamide Q3 (**1**) and nobilamide R3 (**2**), and one known heptapeptide, **3**. Their structures, including absolute configuration, were determined by comprehensive spectroscopic methods combined with Marfey’s analysis. Genome sequencing and bioinformatic analysis revealed that the *nbl_BS_* BGC was proposed to be responsible for the biosynthesis of **1**–**3**. Based on the substrate specificity of A domains and the function of C domains, a biosynthetic pathway for **1**–**3** was proposed. Compounds **1**–**3** exhibited anti-MDR bacterial activities against the Gram-positive MDR strain *S. aureus* CCARM 3090 with MIC values ranging from 3.25 to 25 μg/mL. Collectively, these findings make contributions to the understanding of this peptide class, serving as a foundation for further semi-synthetic studies. The successful identification and characterization of these bioactive heptapeptides underscore the significance of exploring cold-seep ecosystems as a rich and relatively untapped source of natural products with medical potential.

## Figures and Tables

**Table 1:** ^1^H NMR (600 MHz) and ^13^C NMR (150 MHz) data in DMSO-*d*_6_ for compounds **1**–**3**.

		1		2		3	
	Pos.	*δ*_C_	*δ*_H_ (*J* in Hz)	*δ*_C_	*δ*_H_ (*J* in Hz)	*δ*_C_	*δ*_H_ (*J* in Hz)
Ac	1	168.7		168.6		168.9	
	2	22.4	1.96 (3H, s)	22.3	1.77 (3H, s)	22.4	1.88 (3H, s)
Phe	1	170.6		171.6		170.8	
	2	52.2	4.64 (1H, m)	53.2	4.59 (1H, dt, *J* = 8.6, 6.2 Hz)	52.8	4.58 (1H, m)
	3	38.0	2.71 (1H, dd, *J* = 13.5, 4.7 Hz)	38.8	2.62 (1H, dd, *J* = 13.4, 9.2 Hz)	37.6	2.70 (1H, dd, *J* = 7.0, 13.7 Hz)
			2.92 (1H, dd, *J* = 13.5, 4.3 Hz)		2.81 (1H, dd, *J* = 13.4, 6.2 Hz)		2.93 (1H, dd, *J* = 4.7, 13.7 Hz)
	4	136.3		137.3		137.0	
	5	130.3	7.01 (1H, d, *J* = 7.3 Hz)	128.0	7.17~7.18 (1H, m)	127.6	7.14–7.17 (1H, m)
	6	127.4	7.09 (1H, t, *J* = 7.4 Hz)	129.1	7.24 (1H, m)	128.0	7.23–7.24 (1H, m)
	7	126.0	7.17 (1H, t, *J* = 7.2 Hz)	126.2	7.18 (1H, m)	129.8	7.10 (1H, s)
	8	127.4	7.09 (1H, t, *J* = 7.4 Hz)	129.1	7.24 (1H, m)	128.0	7.23–7.24 (1H, m)
	9	130.3	7.01 (1H, d, *J* = 7.3 Hz)	128.0	7.17~7.18 (1H, m)	127.6	7.14–7.17 (1H, m)
	NH		8.56 (1H, br s)		7.76 (1H, s)		7.48 (1H, s)
Leu	1	171.6		172.1		171.9	
	2	52.6	3.91 (1H, m)	52.2	3.87 (1H, q, *J* = 7.0 Hz)	52.0	4.06 (1H, m)
	3	39.3	1.11 (1H, m)	39.6	1.04 (1H, m)	40.1	1.19 (1H, m)
			1.34 (1H, m)		1.16 (1H, m)		1.27 (1H, m)
	4	23.2	0.71 (1H, m)	23.4	0.81 (1H, m)	23.6	1.04 (1H, m)
	5	23.3	0.62 (3H, d, *J* = 6.0 Hz)	22.4	0.58 (3H, d, *J* = 6.5 Hz)	22.4	0.68 (3H, d, *J* = 7.7 Hz)
	6	22.0	0.78 (3H, d, *J* = 5.7 Hz)	22.5	0.71 (3H, d, *J* = 6.5 Hz)	22.4	0.78 (3H, d, *J* = 7.7 Hz)
			8.41 (1H, br s)		8.18 (1H, d, *J* = 7.0 Hz)		8.19 (1H, s)
Phe	1	171.2		171.4		171.4	
	2	54.0	4.66 (1H, m)	54.6	4.40 (1H, ddd, *J* = 11.8, 8.5, 4.1 Hz)	54.4	4.54 (1H, m)
	3	37.0	2.97 (1H, d, *J* = 12.8 Hz)	36.9	2.92 (1H, dd, *J* = 13.3, 11.5 Hz)	37.1	2.88 (1H, t, *J* = 12.8 Hz)
			3.25 (1H, m)		3.15 (1H, dd, *J* = 13.3, 4.1 Hz)		3.16 (1H, dd, *J* = 14.1, 4.5 Hz)
	4	138.2		138.0		137.9	
	5	129.2	7.28 (1H, t, *J* = 7.2 Hz)	128.0	7.23 (1H, m)	126.1	7.17–7.20 (1H, m)
	6	128.0	7.25 (1H, t, *J* = 7.4 Hz)	129.1	7.23~7.24 (1H, m)	129.1	7.25–7.27 (1H, m)
	7	126.2	7.18 (1H, t, *J* = 7.2 Hz)	126.2	7.16 (1H, m)	129.8	7.11 (1H, s)
	8	128.0	7.25 (1H, t, *J* = 7.4 Hz)	129.1	7.23~7.24 (1H, m)	129.1	7.25–7.27 (1H, m)
	9	129.2	7.28 (1H, t, *J* = 7.2 Hz)	128.0	7.23 (1H, m)	126.2	7.17–7.20 (1H, m)
	NH		9.52 (1H, br s)		9.32 (1H, s)		9.31 (1H, s)
Thr	1	167.2		167.4		167.4	
	2	58.5	3.78 (1H, d, *J* = 5.2 Hz)	58.0	4.15 (1H, dd, *J* = 6.5, 1.2 Hz)	58.1	3.91 (1H, m)
	3	72.0	3.75 (1H, m)	73.2	4.84 (1H, q, *J* = 6.5 Hz)	72.5	3.42 (1H, q, *J* = 5.3 Hz)
	4	16.6	1.10 (3H, d, *J* = 6.2 Hz)	16.9	1.39 (3H, d, *J* = 5.9 Hz)	16.9	1.18 (3H, d, *J* = 5.9 Hz)
	NH		7.94 (1H, s)		8.47 (1H, d, *J* = 6.5 Hz)		8.14 (1H, s)
Val	1	170.6		170.8		170.7	
	2	61.3	3.94 (1H, m)	61.2	3.97 (1H, t, *J* = 10.3 Hz)	61.1	3.94 (1H, t, *J* = 10.3 Hz)
	3	29.6	2.04 (1H, m)	29.2	2.02 (1H, m)	29.1	1.97 (1H, m);
	4	19.2	0.82 (3H, d, *J* = 6.3 Hz)	19.1	0.82 (3H, d, *J* = 6.6 Hz)	19.1	0.80 (3H, d, *J* = 6.7 Hz)
	5	19.0	0.87 (3H, d, *J* = 6.5 Hz)	19.1	0.88 (3H, d, *J* = 6.6 Hz)	19.1	0.85 (3H, d, *J* = 6.6 Hz)
	NH		8.36 (1H, d, *J* = 9.3 Hz)		8.26 (1H, s)		8.09 (1H, s)
Ala	1	170.3		170.2		170.1	
	2	50.5	4.26 (1H, m)	40.0	4.31 (1H, dt, *J* = 16.1, 7.3 Hz)	49.9	4.27 (1H, dt, *J* = 7.6, 16.0 Hz)
	3	17.5	1.38 (3H, d, *J* = 7.3 Hz)	17.6	1.37 (3H, d, *J* = 7.3 Hz)	17.6	1.34 (3H, d, *J* = 7.3 Hz)
	NH		9.13 (1H, br s)		9.11 (1H, s)		8.91 (1H, s)
Dhb	1	164.0		163.8		163.5	
	2	125.5		125.8		125.7	
	3	134.4	6.63 (1H, q, *J* = 7.0 Hz)	134.2	6.76 (1H, q, *J* = 7.2 Hz)	133.9	6.65 (1H, q, *J* = 7.2 Hz)
	4	15.1	1.57 (3H, d, *J* = 7.0 Hz)	15.0	1.61 (3H, d, *J* = 7.2 Hz)	14.9	1.60 (3H, d, *J* = 7.2 Hz)
	NH		8.86 (1H, s)		8.77 (1H, br s)		8.69 (1H, br s)

**Table 2:** The MIC values of compounds **1**–**3** against different MDR bacterial strains (MIC, μg/mL).

	1	2	3	Tet.
Gram-positive				
*S*. *aureus* CCARM 3090	25	3.25	6.25	6.25
*E*. *faecalis* CCARM 5172	>25	>25	>25	>25
*E*. *faecium* CCARM 5203	>25	>25	>25	0.02
*M*. *luteus* ML01	>25	>25	>25	12.5
Gram-negative				
*E*. *coli* CCARM 1009	>25	>25	>25	0.39
*S*. *typhimurium* CCARM 8250	>25	>25	>25	>25
*A*. *baumannii* ATCC 19606	>25	>25	>25	>25
*K*. *pneumoniae* ATCC 13883	25	>25	>25	12.5
*P*. *aeruginosa* 15690	25	>25	>25	6.25

Tet: tetracycline as a positive control.

## Data Availability

All data is contained within this article and the Supplementary Materials.

## Supplementary Materials

The following supporting information can be downloaded at: https://www.mdpi.com/article/10.3390/molecules31030547/s1, Table S1: 16s rRNA sequence of B. subtilis 4-L-22; Table S2: BGCs in the genome of B. subtilis 4-L-22; Table S3: Functional annotation of genes in nob gene cluster; Figure S1: The neighbor-joining phylogenetic tree of B. subtilis 4-L-22 based on 16S rRNA sequence; Figure S2: Optimization of the fermentation time for B. subtilis 4-L-22; Figure S3: The anti-MDR bacterial activity of crude extracts of B. subtilis 4-L-22; Figure S4: UV spectrum of nobilamide Q3 (**1**); Figure S5: HR-ESI-MS spectrum of nobilamide Q3 (**1**); Figures S6–S11: The 1D and 2D NMR spectrum of nobilamide Q3 (**1**) in DMSO-d6; Figure S12: MS2 analysis of nobilamide Q3 (**1**); Figure S13: UV spectrum of nobilamide R3 (**2**); Figure S14: HR-ESI-MS spectrum of nobilamide R3 (**2**); Figures S15–S20: The 1D and 2D NMR spectrum of nobilamide R3 (**2**) in DMSO-d6; Figure S21: UV spectrum of A-3302-B (**3**); Figure S22: HR-ESI-MS spectrum of A-3302-B (**3**); Figures S23–S28: The 1D and 2D NMR spectrum of A-3302-B (**3**) in DMSO-d6; Figure S29: HPLC profile of FDAA-derivatives of nobilamide Q3 (**1**) and standard amino acid; Figure S30: HPLC profile of FDAA-derivatives of nobilamide R3 (**2**) and standard amino acid; Figure S31: HPLC profile of FDAA-derivatives of A-3302-B (**3**) and standard amino acid.
