# Supplementary material for: Genome-Driven Discovery of Anti-MDR Bacterial Heptapeptides from a Cold-Seep-Derived Bacillus Strain
Source: Molecules. 2026 Feb 4;31(3):547. doi: 10.3390/molecules31030547 (PMC12899971; doi:10.3390/molecules31030547)

Supporting Information

Genome-Driven Discovery of Anti-MDR Bacterial Heptapeptides from a Cold-Seep-Derived *Bacillus* Strain

Hongcheng Li [1,†], Yongmeng Cheng [1,†], Kaishuai Xing [1], Wenli Li [1,2,3,4] and Fei Xiao [1,*]

[1] Key Laboratory of Marine Drugs, Ministry of Education of China, School of Medicine and Pharmacy, Ocean
University of China, Qingdao 266003, China; lihongcheng@stu.ouc.edu.cn (H.L.); 18945124627@163.com (Y.C.); xingkaishuai566@126.com (K.X.); liwenli@ouc.edu.cn (W.L.)

[2] Laboratory for Marine Drugs and Bioproducts, Qingdao Marine Science and Technology Center,
Qingdao 266237, China

[3] State Key Laboratory for Crop Stress Resistance and High-Efficiency Production, College of Chemistry & Pharmacy, Northwest A&F University, Yangling 712100, China

[4] Shaanxi Key Laboratory of Natural Products & Chemical Biology, College of Chemistry & Pharmacy, Northwest A&F University, Yangling 712100, China

* Correspondence: xiaofei3450@ouc.edu.cn

† These authors contributed equally to this work.

Table of contents Page

Table S1. 16s rRNA sequence of *B. subtilis* 4-L-22 S4

Table S2. BGCs in the genome of *B. subtilis* 4-L-22 S5

Table S3. Functional annotation of genes in *nob* gene cluster S6

Figure S1. The neighbor-joining phylogenetic tree of *B. subtilis* 4-L-22 based on 16S rRNA sequence. S7

Figure S2. Optimization of the fermentation time for *B. subtilis* 4-L-22. S8

Figure S3 The anti-MDR bacterial activity of crude extracts of *B. subtilis* 4-L-22. S9

Figure S4. UV spectrum of nobilamide Q3 (1) S10

Figure S5. HR-ESI-MS spectrum of nobilamide Q3 (1) S11

Figure S6. ^{1}H NMR spectrum of nobilamide Q3 (1) in DMSO-d_6 S12

Figure S7. ^{13}C NMR spectrum of nobilamide Q3 (1) in DMSO-d_6 S13

Figure S8. HSQC spectrum of nobilamide Q3 (1) in DMSO-d_6 S14

Figure S9. ^{1}H-^{1}H COSY spectrum of nobilamide Q3 (1) in DMSO-d_6 S15

Figure S10. HMBC spectrum of nobilamide Q3 (1) in DMSO-d_6 S16

Figure S11. NOESY spectrum of nobilamide Q3 (1) in DMSO-d_6 S17

Figure S12. MS^2 analysis of nobilamide Q3 (1) S18

Figure S13. UV spectrum of nobilamide R3 (2) S19

Figure S14. HR-ESI-MS spectrum of nobilamide R3 (2) S20

Figure S15. ^{1}H NMR spectrum of nobilamide R3 (2) in DMSO-d_6 S21

Figure S16. ^{13}C NMR spectrum of nobilamide R3 (2) in DMSO-d_6 S22

Figure S17. HSQC spectrum of nobilamide R3 (2) in DMSO-d_6 S23

Figure S18. ^{1}H-^{1}H COSY spectrum of nobilamide R3 (2) in DMSO-d_6 S24

Figure S19. HMBC spectrum of nobilamide R3 (2) in DMSO-d_6 S25

Figure S20. NOESY spectrum of nobilamide R3 (2) in DMSO-d_6 S26

Figure S21. UV spectrum of A-3302-B (3) S27

Figure S22. HR-ESI-MS spectrum of A-3302-B (3) S28

Figure S23. ^{1}H **NMR spectrum of A-3302-B (3) in DMSO-*d*$_6$** **S29**

Figure S24. ^{13}C **NMR spectrum of A-3302-B (3) in DMSO-*d*$_6$** **S30**

Figure S25. HSQC spectrum of A-3302-B (3) in DMSO-*d*$_6$ **S31**

Figure S26. ^{1}H-^{1}H **COSY spectrum of A-3302-B (3) in DMSO-*d*$_6$** **S32**

Figure S27. HMBC spectrum of A-3302-B (3) in DMSO-*d*$_6$ **S33**

Figure S28. NOESY spectrum of A-3302-B (3) in DMSO-*d*$_6$ **S34**

Figure S29. HPLC profile of FDAA-derivatives of nobilamide Q3 (1) and standard amino acid. **S35**

Figure S30. HPLC profile of FDAA-derivatives of nobilamide R3 (2) and standard amino acid. **S36**

Figure S31. HPLC profile of FDAA-derivatives of A-3302-B (3) and standard amino acid. **S37**

Table S1. 16s rRNA sequence of *B. subtilis* 4-L-22

GACGAACGCTGGCGGCGTGCCTAATACATGCAAGTCGAGCGGACAGATGGGAGCT<br>TGCTCCCTGATGTTAGCGGCGGACGGGTGAGTAACACGTGGGTAACCTGCCTGTAA<br>GACTGGGATAACTCCGGGAAACCGGGGCTAATACCGGATGGTTGTTTGAACCGCA<br>TGGTTCAAACATAAAAGGTGGCTTCGGCTACCACTTACAGATGGACCCGCGGCGC<br>ATTAGCTAGTTGGTGAGGTAACGGCTCACCAAGGCGACGATGCGTAGCCGACCTG<br>AGAGGGTGATCGGCCACACTGGGACTGAGACACGGCCCAGACTCCTACGGGAGGC<br>AGCAGTAGGGAATCTTCCGCAATGGACGAAAGTCTGACGGAGCAACGCCGCGTGA<br>GTGATGAAGGTTTTCGGATCGTAAAGCTCTGTTGTTAGGGAAGAACAAGTACCGTT<br>CGAATAGGGCGGTACCTTGACGGTACCTAACCAGAAAGCCACGGCTAACTACGTG<br>CCAGCAGCCGCGGTAATACGTAGGTGGCAAGCGTTGTCCGGAATTATTGGGCGTA<br>AAGGGCTCGCAGGCGGTTTCTTAAGTCTGATGTGAAAGCCCCCGGCTCAACCGGGG<br>AGGGTCATTGGAAACTGGGGAACTTGAGTGCAGAAGAGGAGAGTGGAATTCCACG<br>TGTAGCGGTGAAATGCGTAGAGATGTGGAGGAACACCAGTGGCGAAGGCGACTCT<br>CTGGTCTGTAACTGACGCTGAGGAGCGAAAGCGTGGGGAGCGAACAGGATTAGAT<br>ACCCTGGTAGTCCACGCCGTAAACGATGAGTGCTAAGTGTTAGGGGGTTTCCGCCC<br>CTTAGTGCTGCAGCTAACGCATTAAGCACTCCGCCTGGGGAGTACGGTCGCAAGAC<br>TGAAACTCAAAGGAATTGACGGGGGCCCGCACAAGCGGTGGAGCATGTGGTTTAA<br>TTCGAAGCAACGCGAAGAACCTTACCAGGTCTTGACATCCTCTGACAATCCTAGAG<br>ATAGGACGTCCCCTTCGGGGGCAGAGTGACAGGTGGTGCATGGTTGTCGTCAGCTC<br>GTGTCGTGAGATGTTGGGTTAAGTCCCGCAACGAGCGCAACCCTTGATCTTAGTTG<br>CCAGCATTCAGTTGGGCACTCTAAGGTGACTGCCGGTGACAAACCGGAGGAAGGT<br>GGGGATGACGTCAAATCATCATGCCCCTTATGACCTGGGCTACACACGTGCTACAA<br>TGGACAGAACAAAGGGCAGCGAAACCGCGAGGTTAAGCCAATCCCACAAATCTG<br>TTCTCAGTTCGGATCGCAGTCTGCAACTCGACTGCGTGAAGCTGGAATCGCTAGTA<br>ATCGCGGATCAGCATGCCGCGGTGAATACGTTCCCGGGCCTTGTACACACCGCCCG<br>TCACACCACGAGAGTTTGTAACACCCGAAGTCGGTGAGGTAACCTTTTAGGAGCCA<br>GCCGCCGAAGGTGGGACAGATGATTGGGGTG

Table S2. BGCs in the genome of *B. subtilis* 4-L-22

Cluster	Locus Tag	Type	Predicted product	Identity
1		NRPS	surfactin	82%
2		terpene		
3		NRPS	paenibacterin	60%
4		TransAT-PKS/PKS-like/T3PKS	bacillaene	100%
5		NRPS/betalactone	fengycin	100%
6		terpene		
7		T3PKS		
8		NRPS	bacillibactin	100%
9		CDPS	pulcherriminic acid	100%
10		sactipeptide	subtilosin	100%
11		other	bacilysin	100%
12		RRE-containing		
13		epipeptide	thailanstatin A	10%

Table S3. Functional annotation of genes in *nob* gene cluster

Locus tag	Putative function	Characterized homolog	Protein identity (%)
ctg1_1554	Cell division ATPase FtsA		
ctg1_1555	Cell division GTPase FtsZ		
ctg1_1556	NRPS	BreC (ATY37608.1)	44%
ctg1_1557	NRPS	BreD (ATY37609.1)	46%
ctg1_1558	Cell division GTPase FtsZ		

Figure S1. The neighbor-joining phylogenetic tree of *B. subtilis* 4-L-22 based on 16S rRNA sequence.

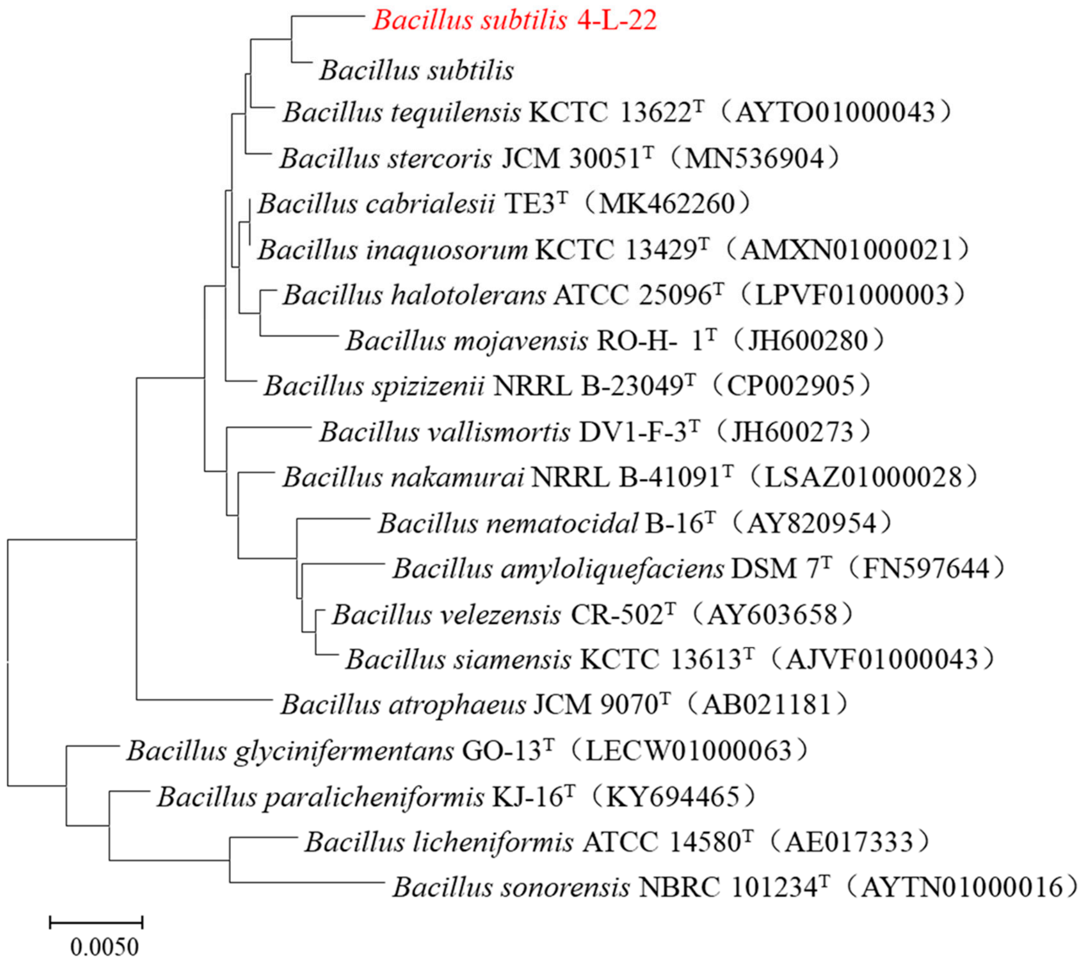

Figure S2. Optimization of the fermentation time for *B. subtilis* 4-L-22. (**A**) The HPLC profile of *B. subtilis* 4-L-22 fermented at different time. (B) The UV absorption of peaks 1-3 in crude extract of *B. subtilis* 4-L-22.

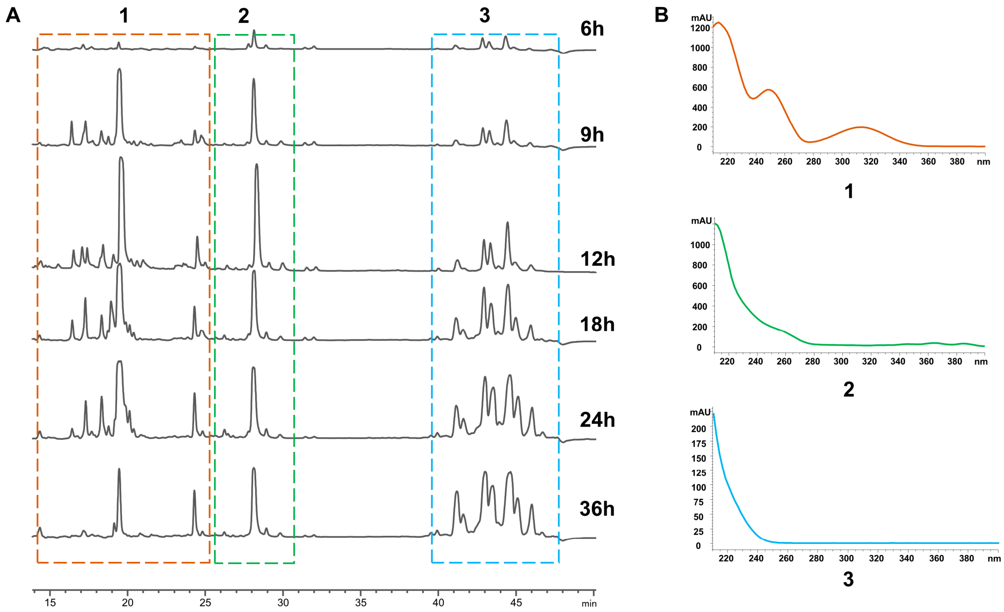

Figure S3. The anti-MDR bacterial activity of crude extracts of *B. subtilis* 4-L-22.

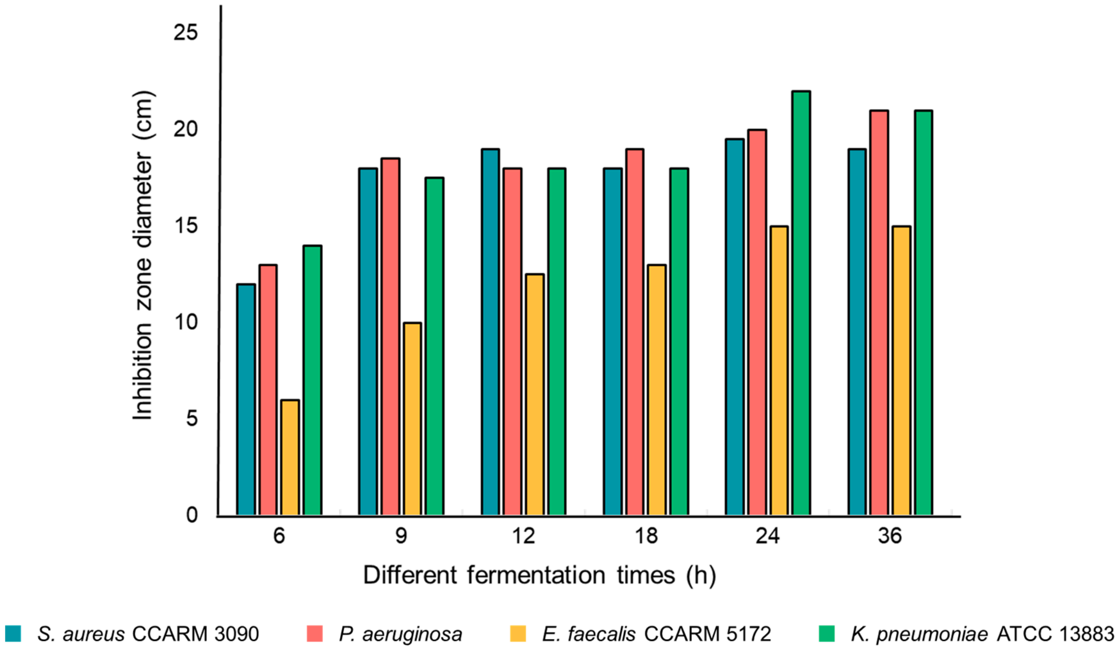

Figure S4. UV spectrum of nobilamide Q3 (**1**)

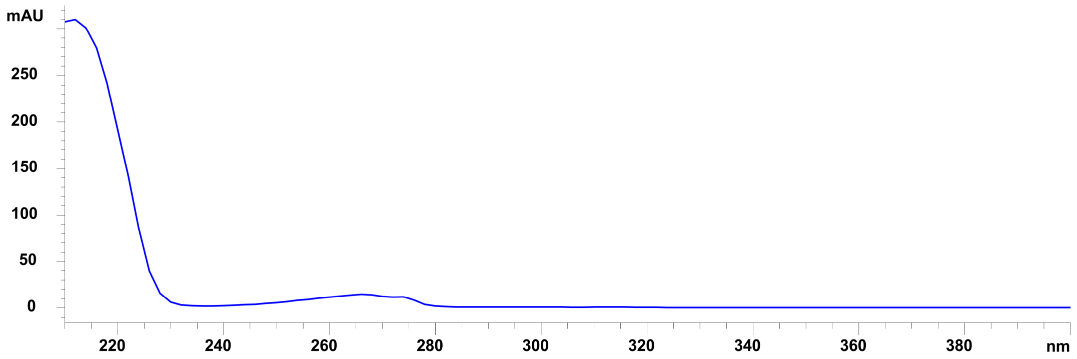

Figure S5. HR-ESI-MS spectrum of nobilamide Q3 (**1**)

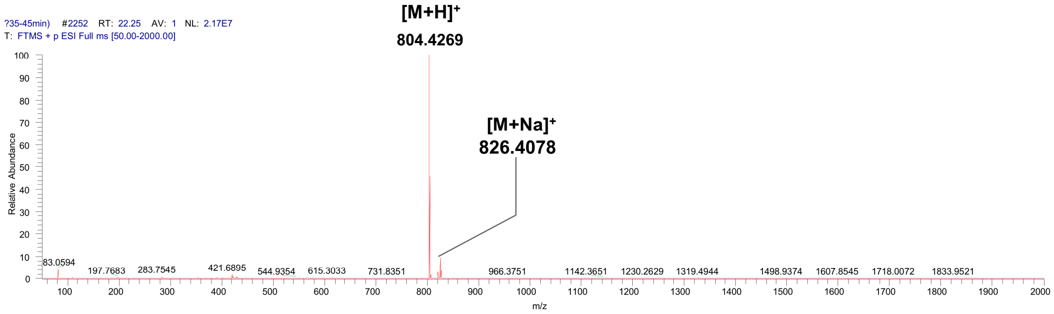

Figure S6. ^{1}H NMR spectrum of nobilamide Q3 (**1**) in DMSO-d_6

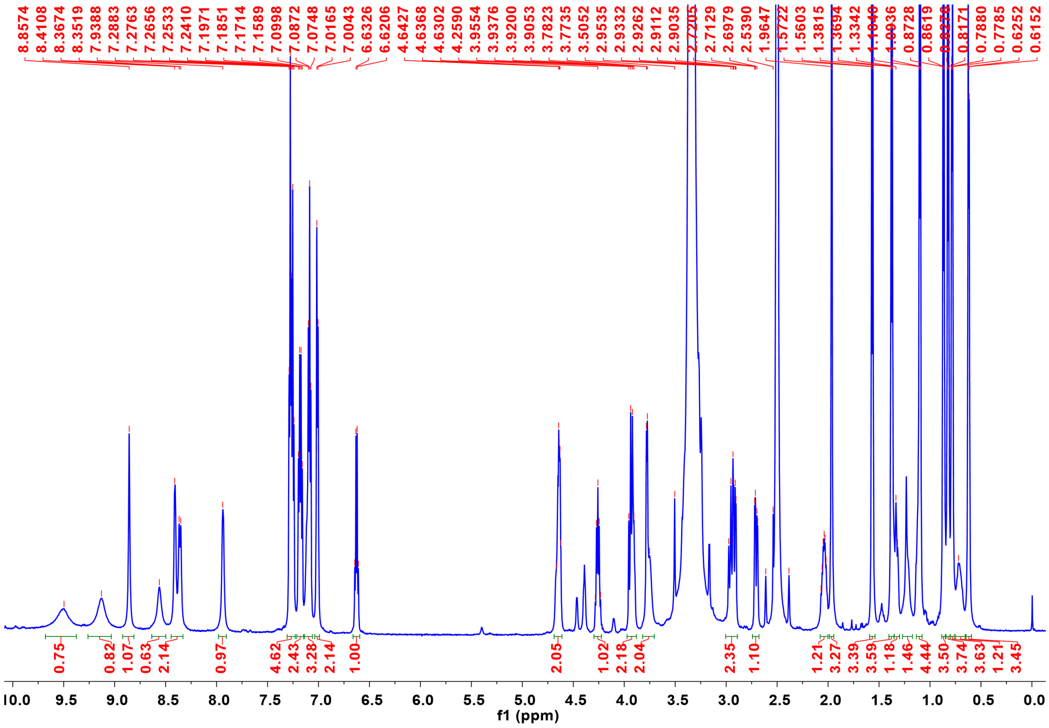

Figure S7. ^{13}C NMR spectrum of nobilamide Q3 (**1**) in DMSO-d_6

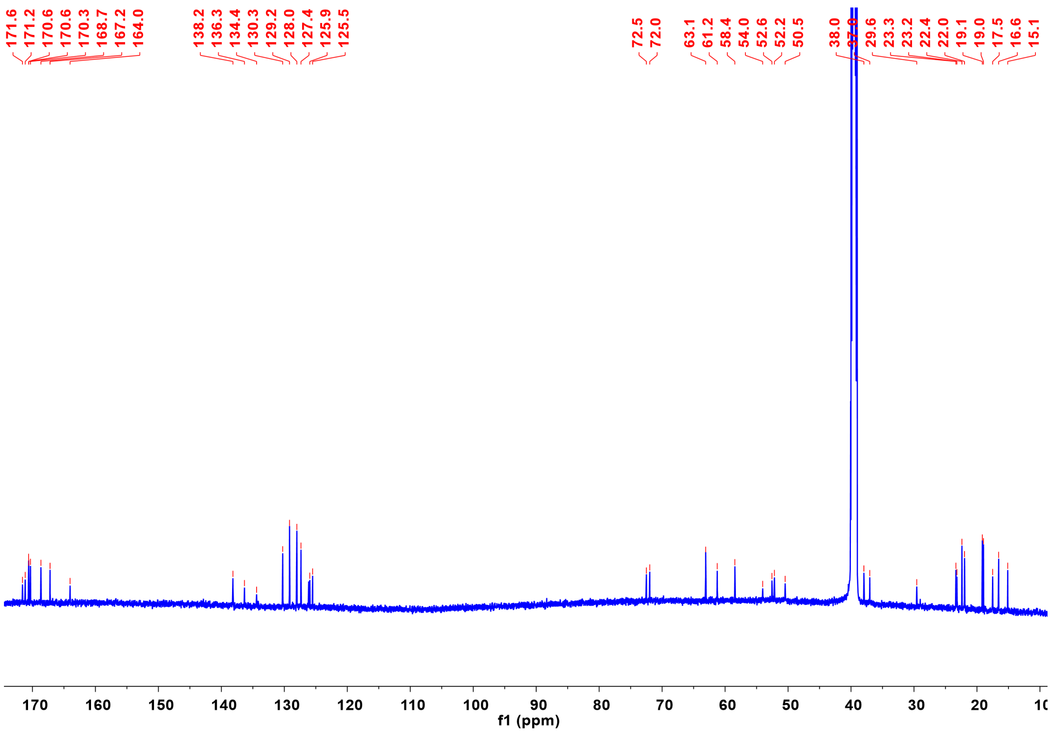

Figure S8. HSQC spectrum of nobilamide Q3 (**1**) in DMSO-*d*6

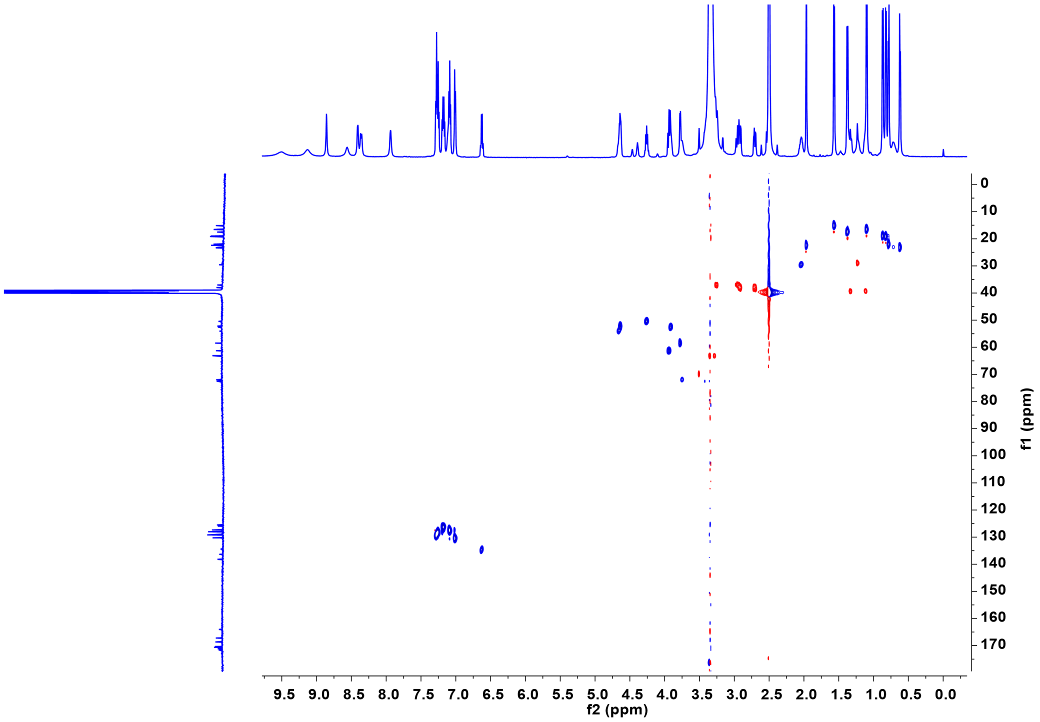

Figure S9. ^{1}H-^{1}H COSY spectrum of nobilamide Q3 (**1**) in DMSO-d_6

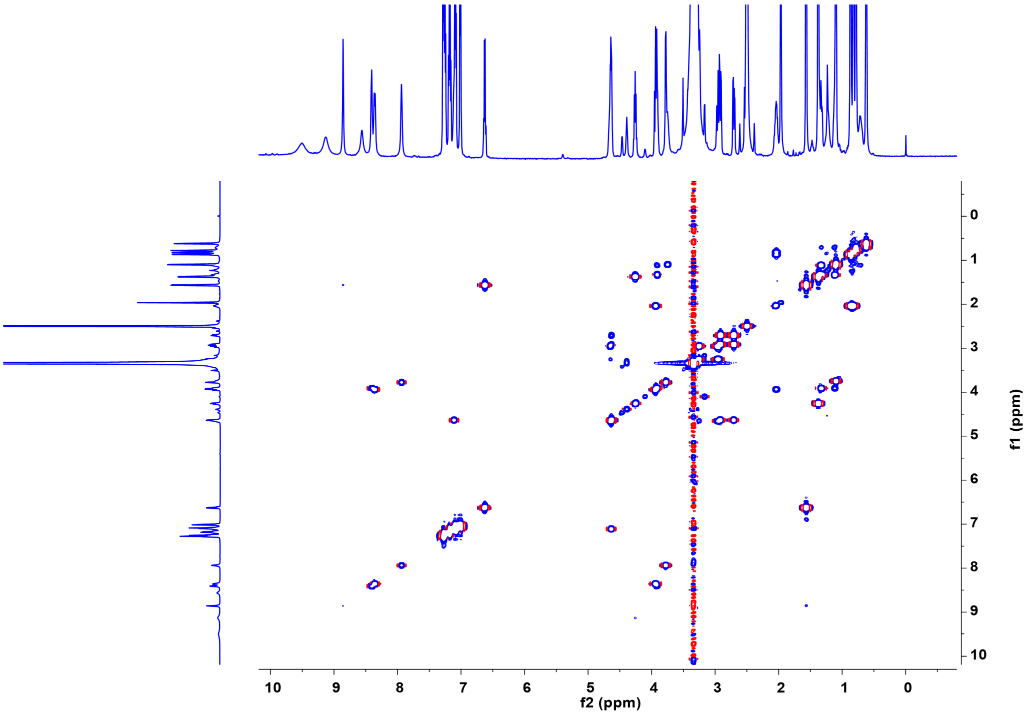

Figure S10. HMBC spectrum of nobilamide Q3 (**1**) in DMSO-d_6

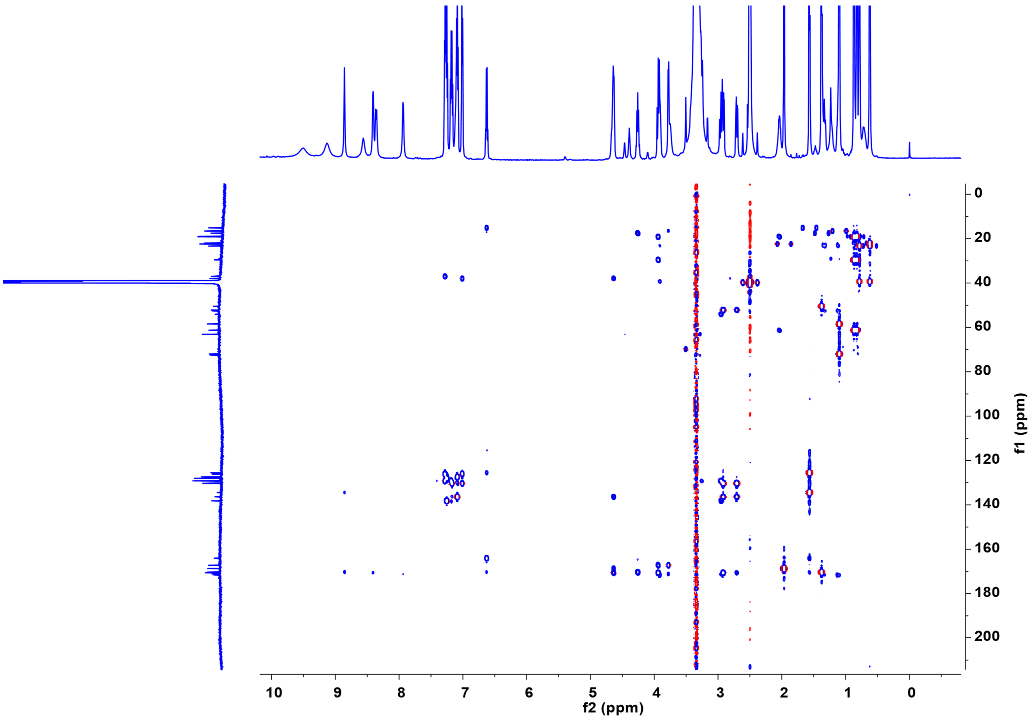

Figure S11. NOESY spectrum of nobilamide Q3 (**1**) in DMSO-d_6

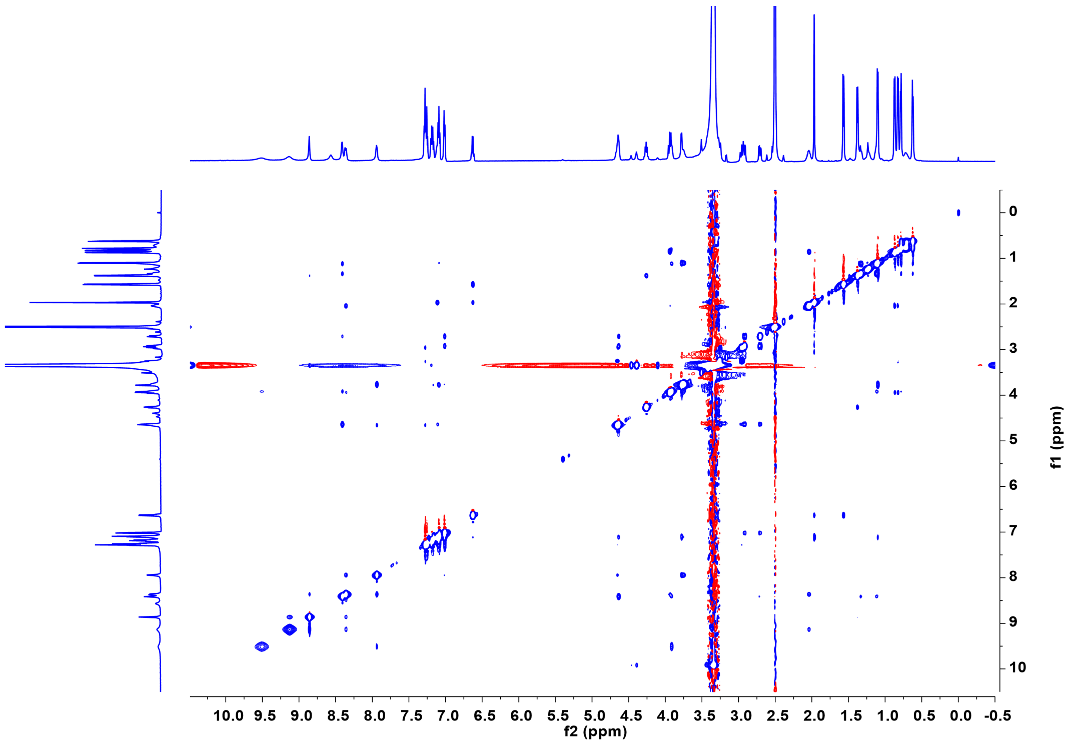

Figure S12. MS^2 analysis of nobilamide Q3 (**1**)

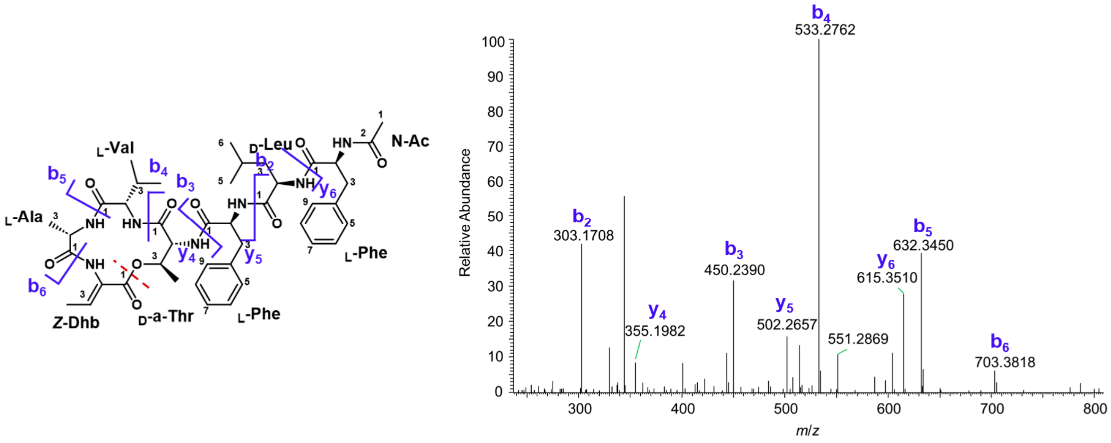

Figure S13. UV spectrum of nobilamide R3 (**2**)

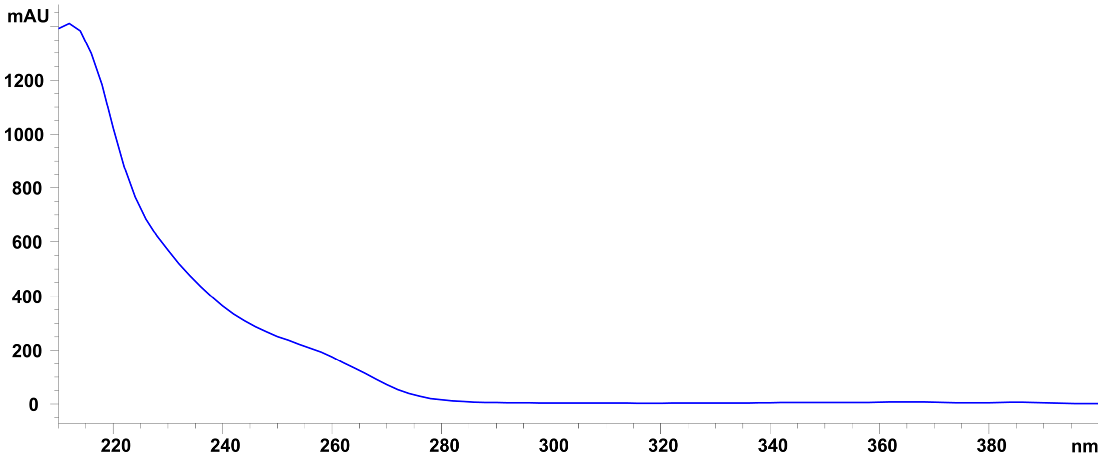

Figure S14. HR-ESI-MS spectrum of nobilamide R3 (**2**)

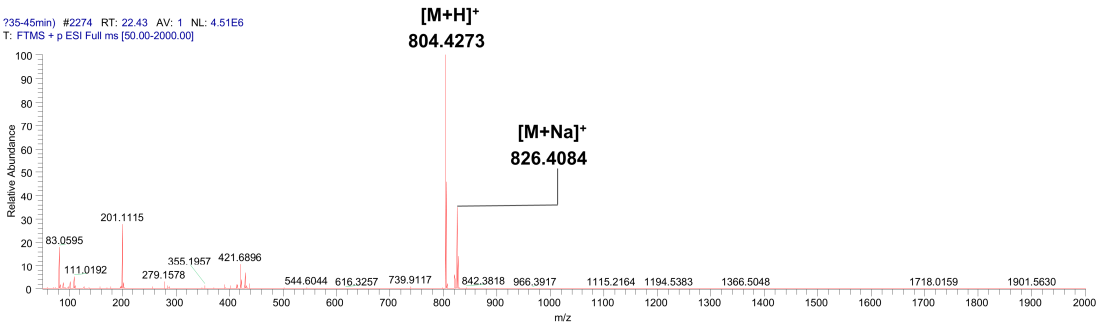

Figure S15. ^{1}H NMR spectrum of nobilamide R3 (**2**) in DMSO-d_6

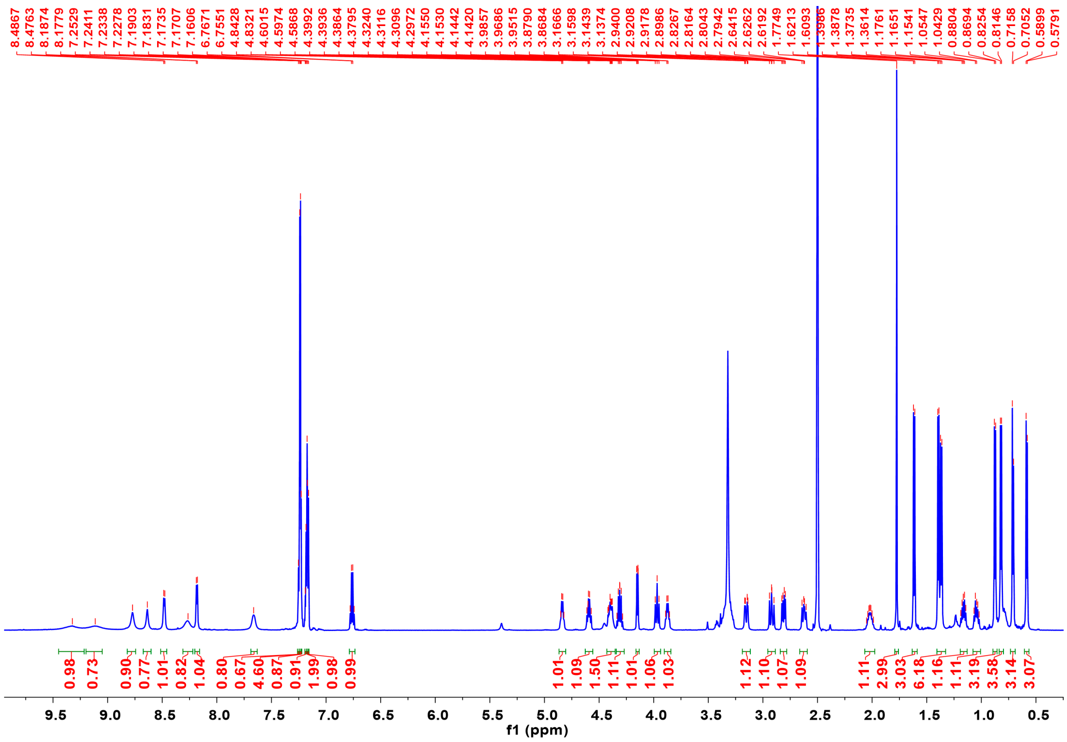

Figure S16. ^{13}C NMR spectrum of nobilamide R3 (**2**) in DMSO-d_6

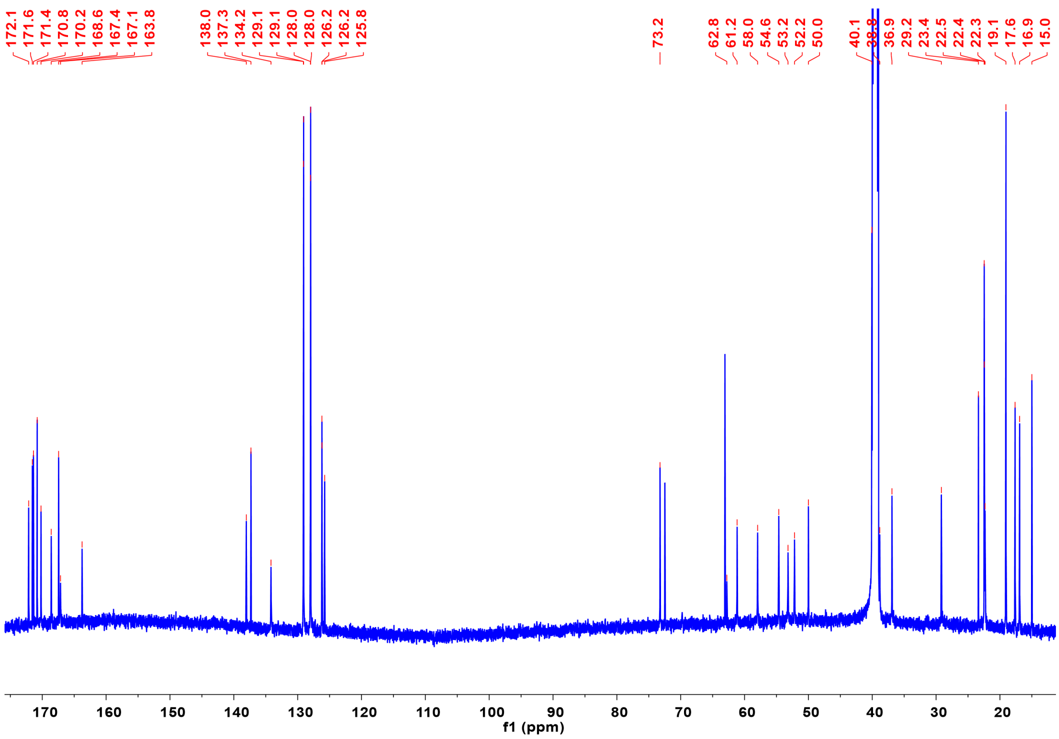

Figure S17. HSQC spectrum of nobilamide R3 (**2**) in DMSO-d_6

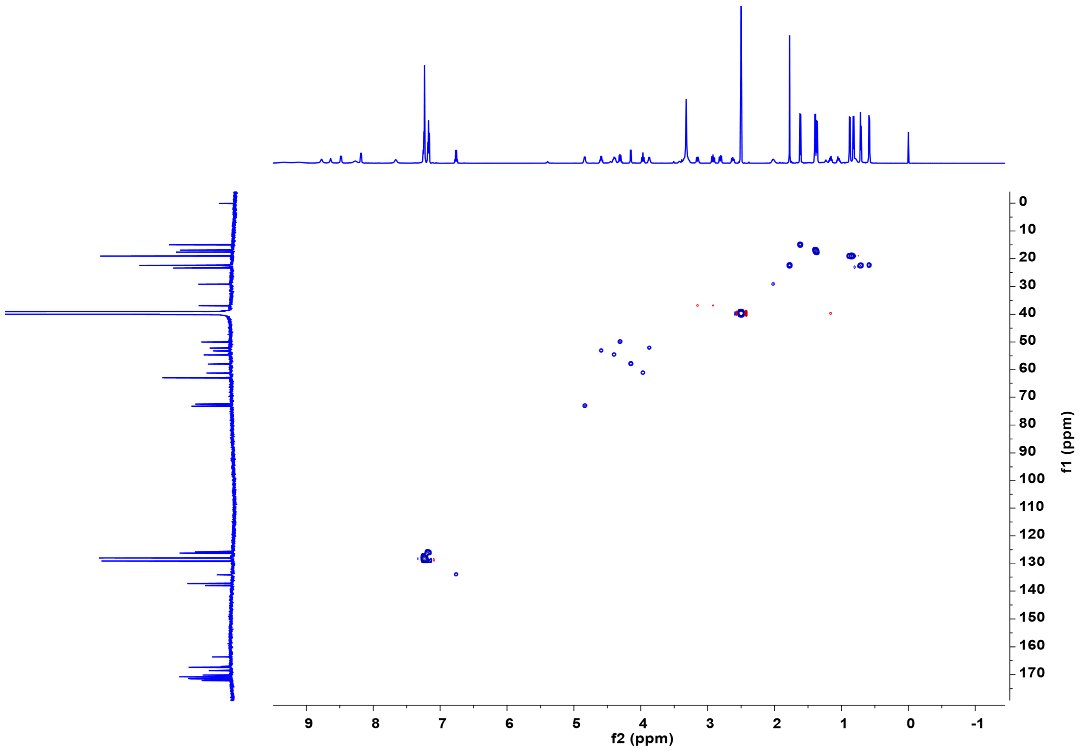

Figure S18. ^1H-^1H COSY spectrum of nobilamide R3 (**2**) in DMSO-d_6

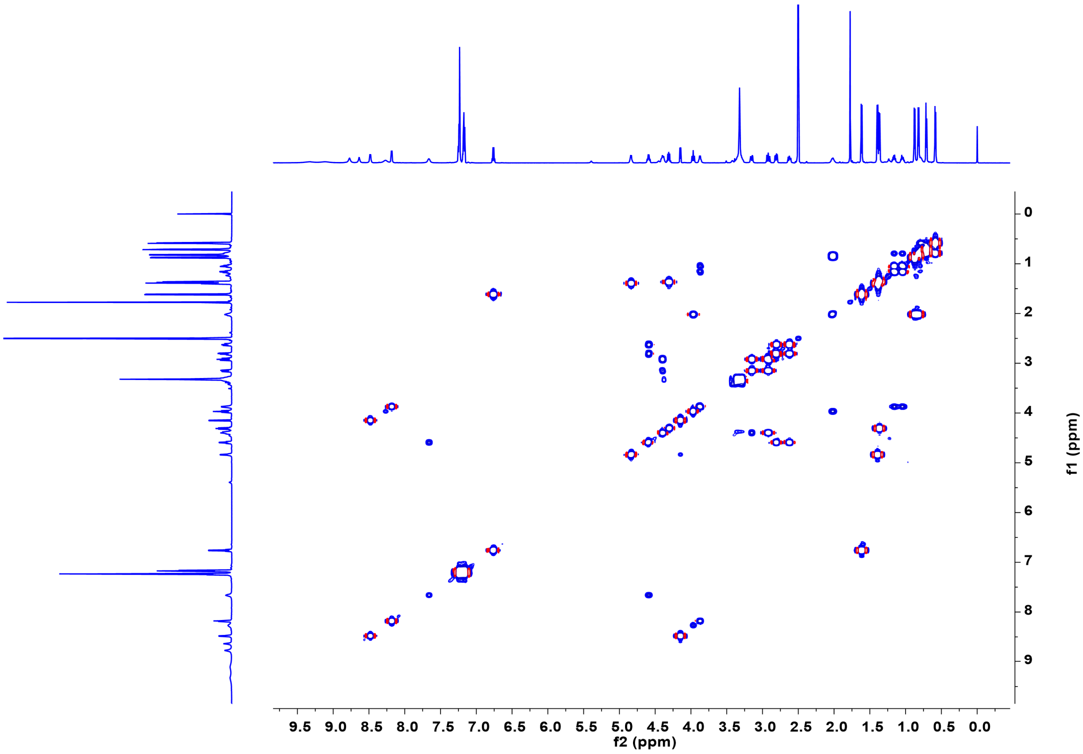

Figure S19. HMBC spectrum of nobilamide R3 (**2**) in DMSO-d_6

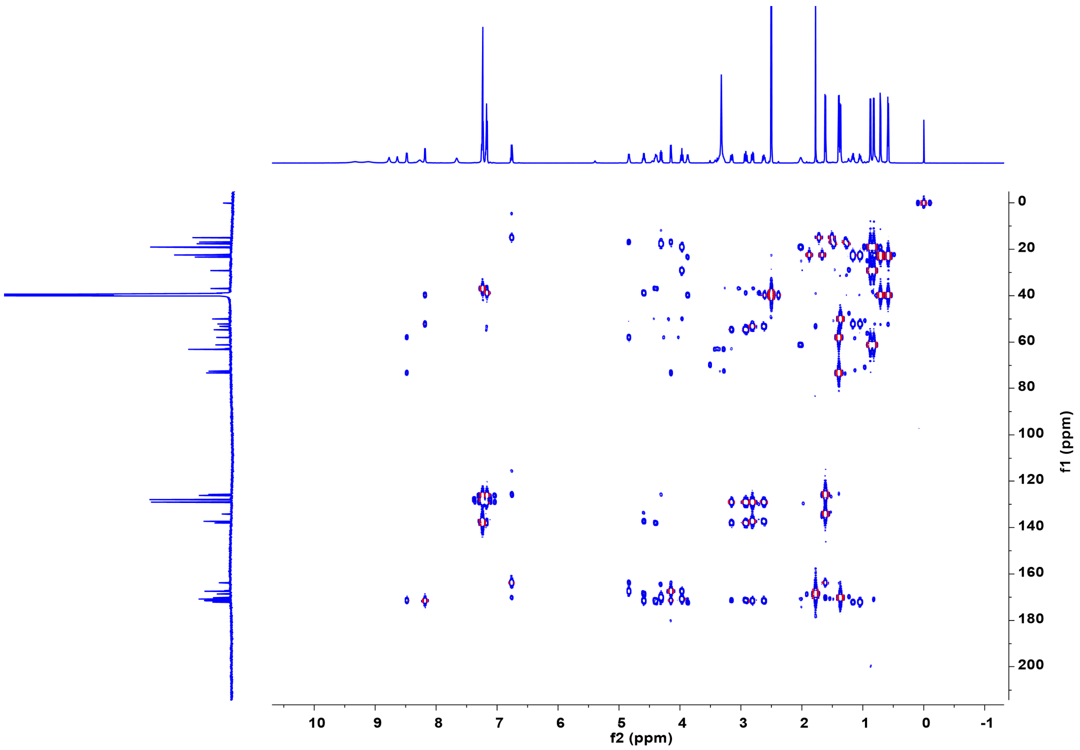

Figure S20. NOESY spectrum of nobilamide R3 (**2**) in DMSO-d_6

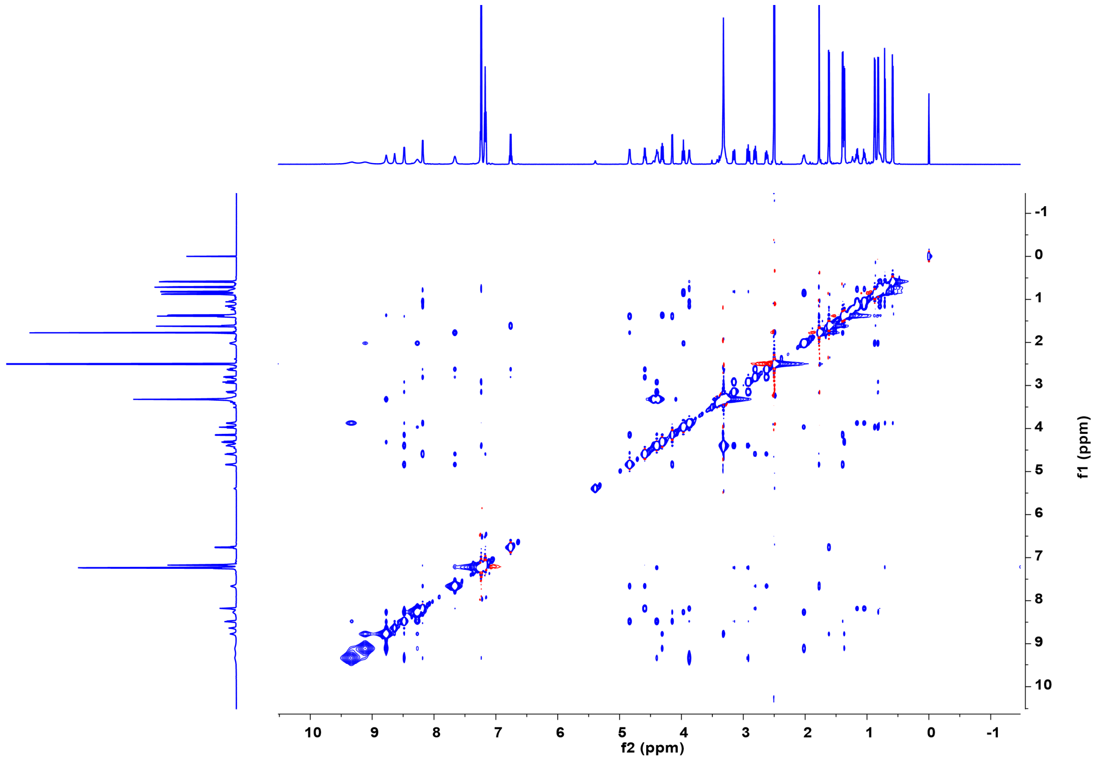

Figure S21. UV spectrum of A-3302-B (**3**)

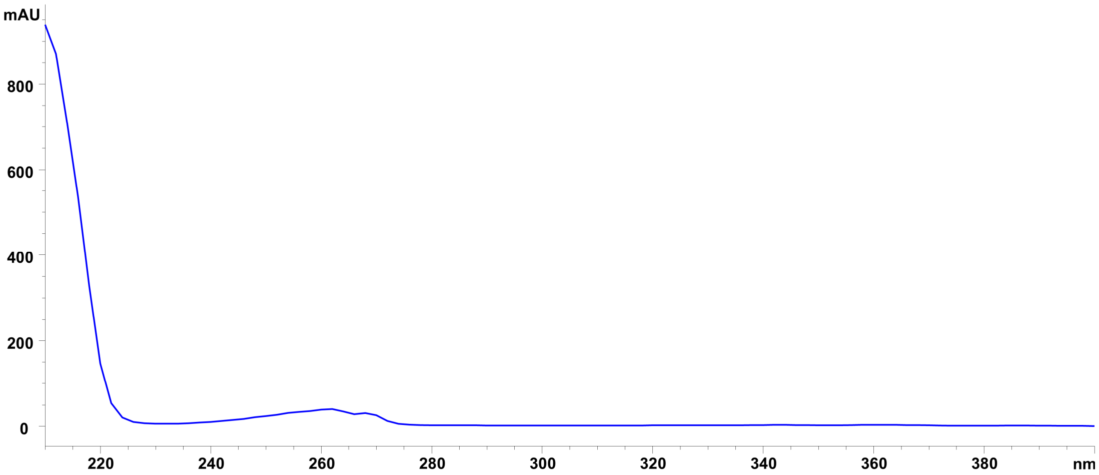

Figure S22. HR-ESI-MS spectrum of A-3302-B (**3**)

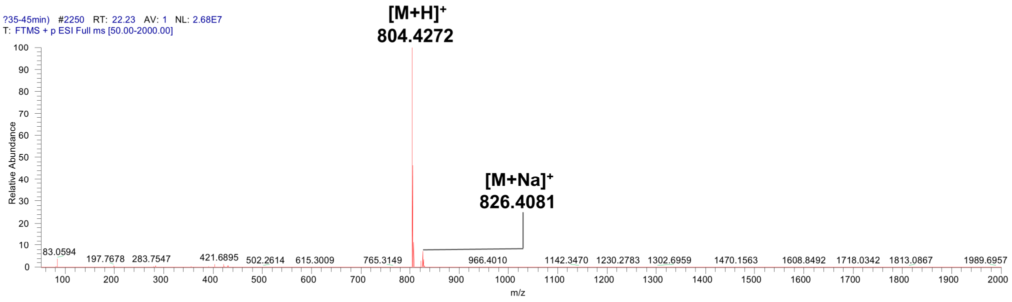

Figure S23. ^{1}H NMR spectrum of A-3302-B (**3**) in DMSO-d_6

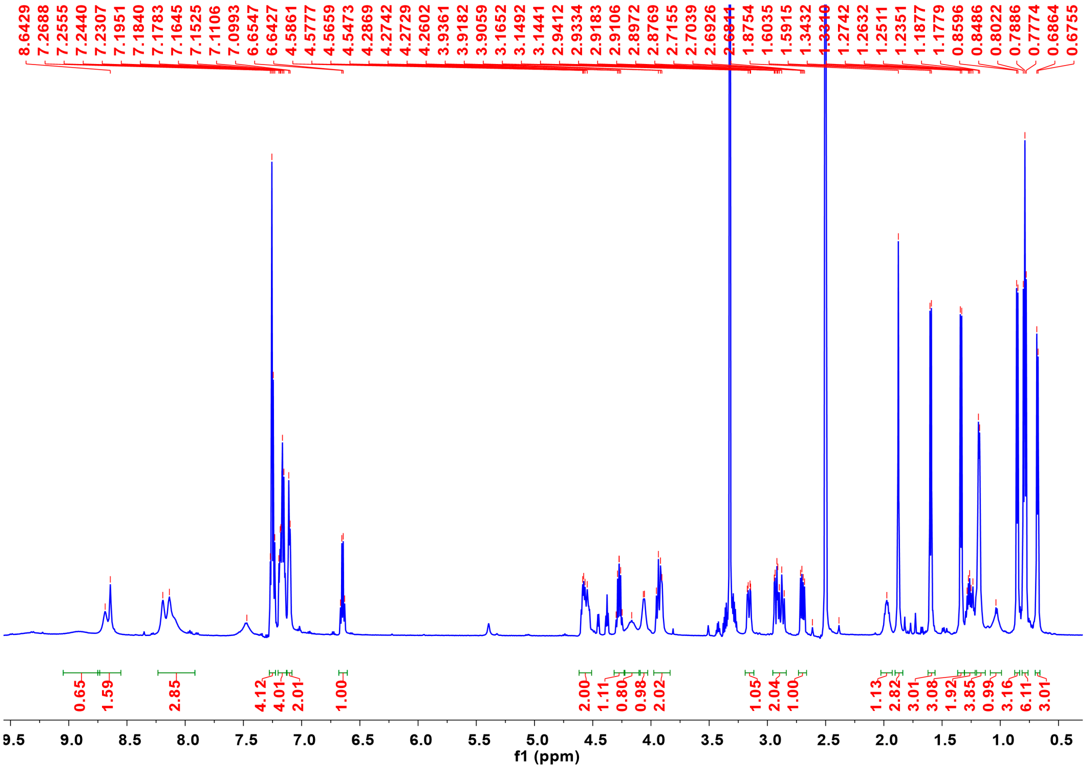

Figure S24. ^{13}C NMR spectrum of A-3302-B (**3**) in DMSO-d_6

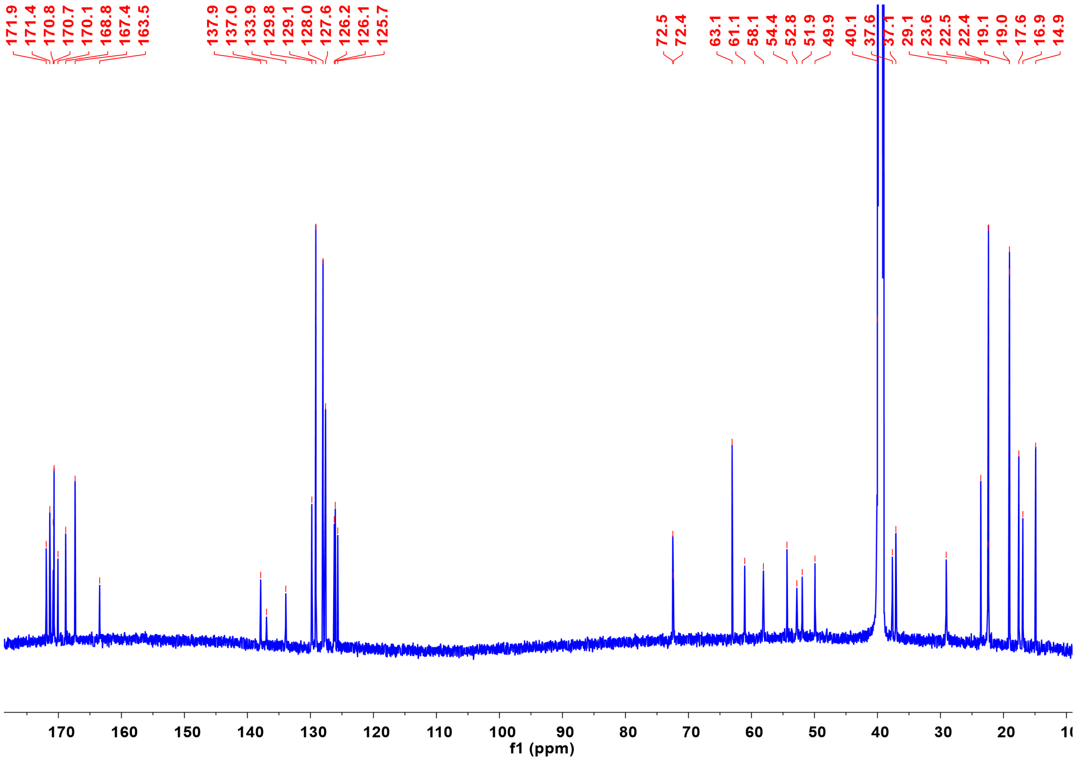

Figure S25. HSQC spectrum of A-3302-B (**3**) in DMSO-d_6

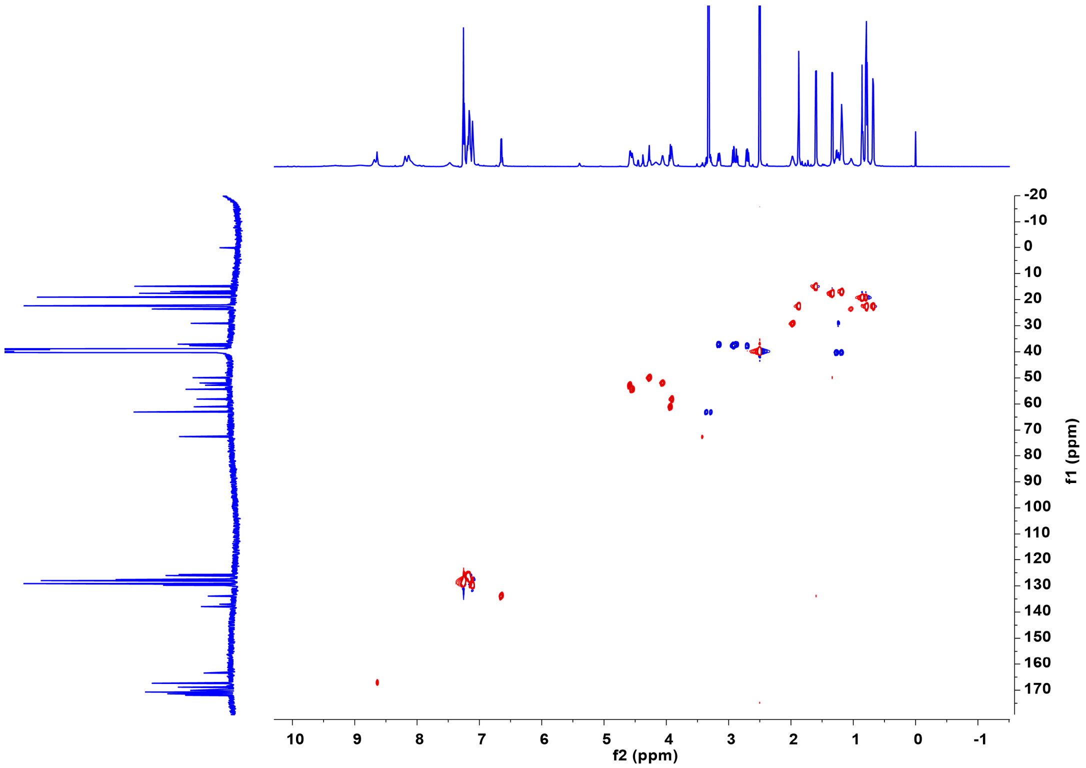

Figure S26. ^{1}H-^{1}H COSY spectrum of A-3302-B (**3**) in DMSO-d_6

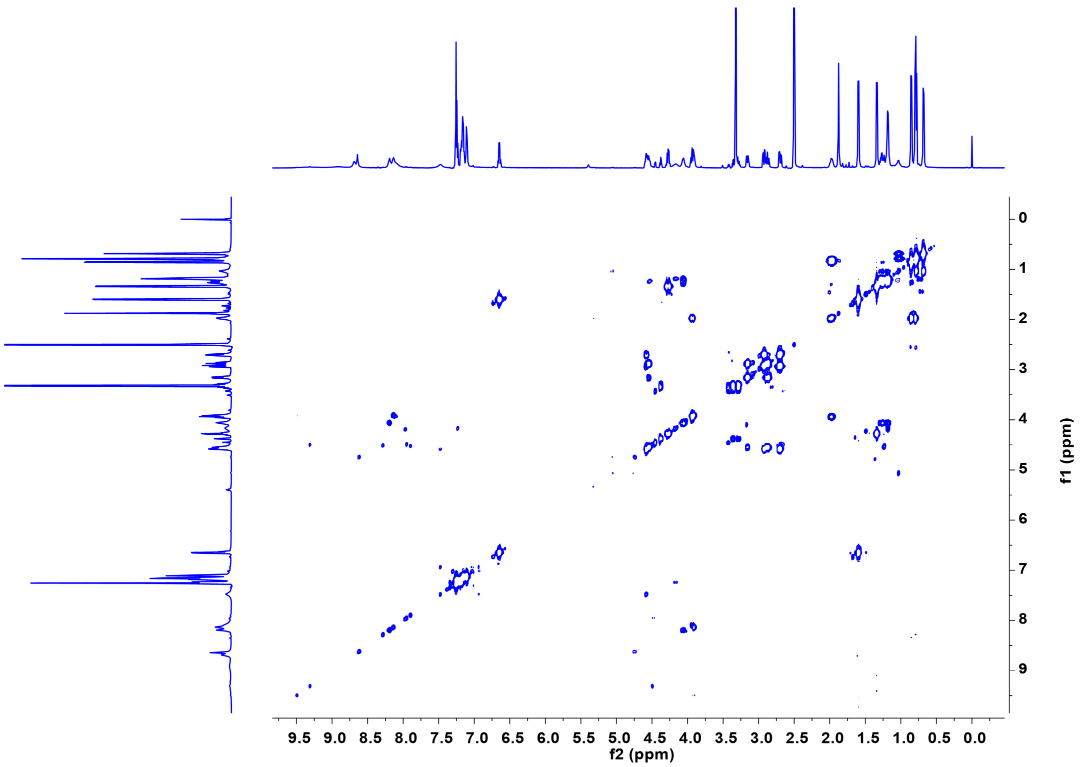

Figure S27. HMBC spectrum of A-3302-B (**3**) in DMSO-d_6

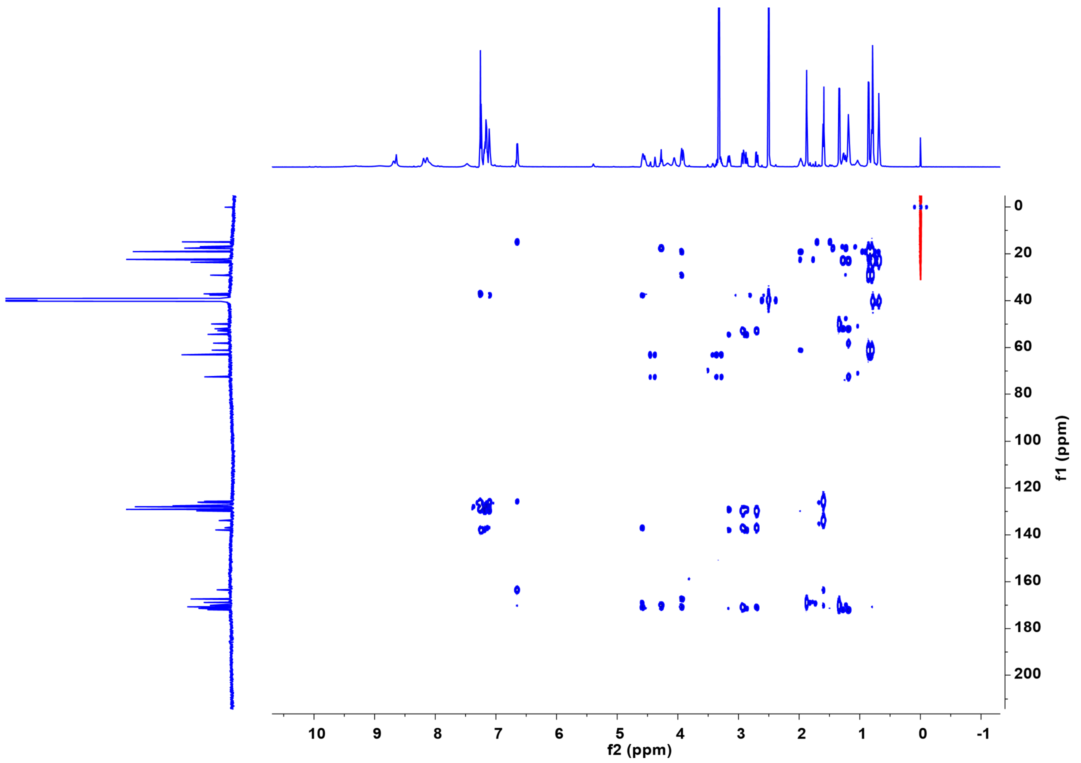

Figure S28. NOESY spectrum of A-3302-B (**3**) in DMSO-d_6

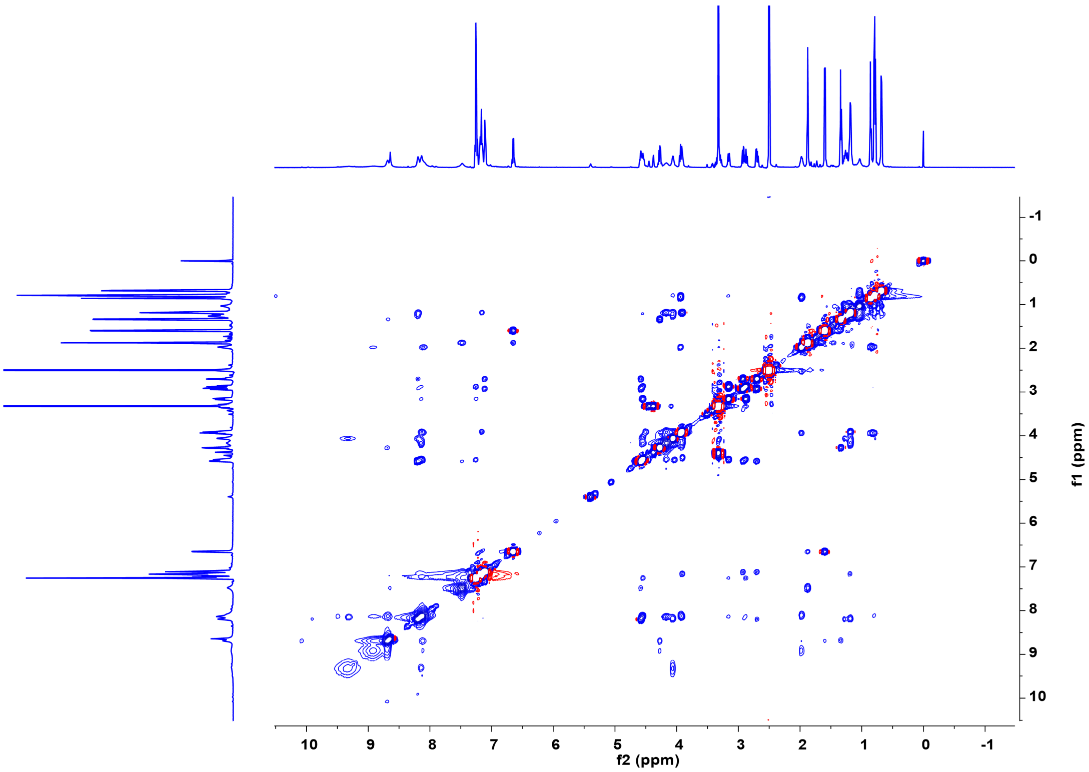

Figure S29. HPLC profile of FDAA-derivatives of nobilamide Q3 (**1**) and standard amino acid.

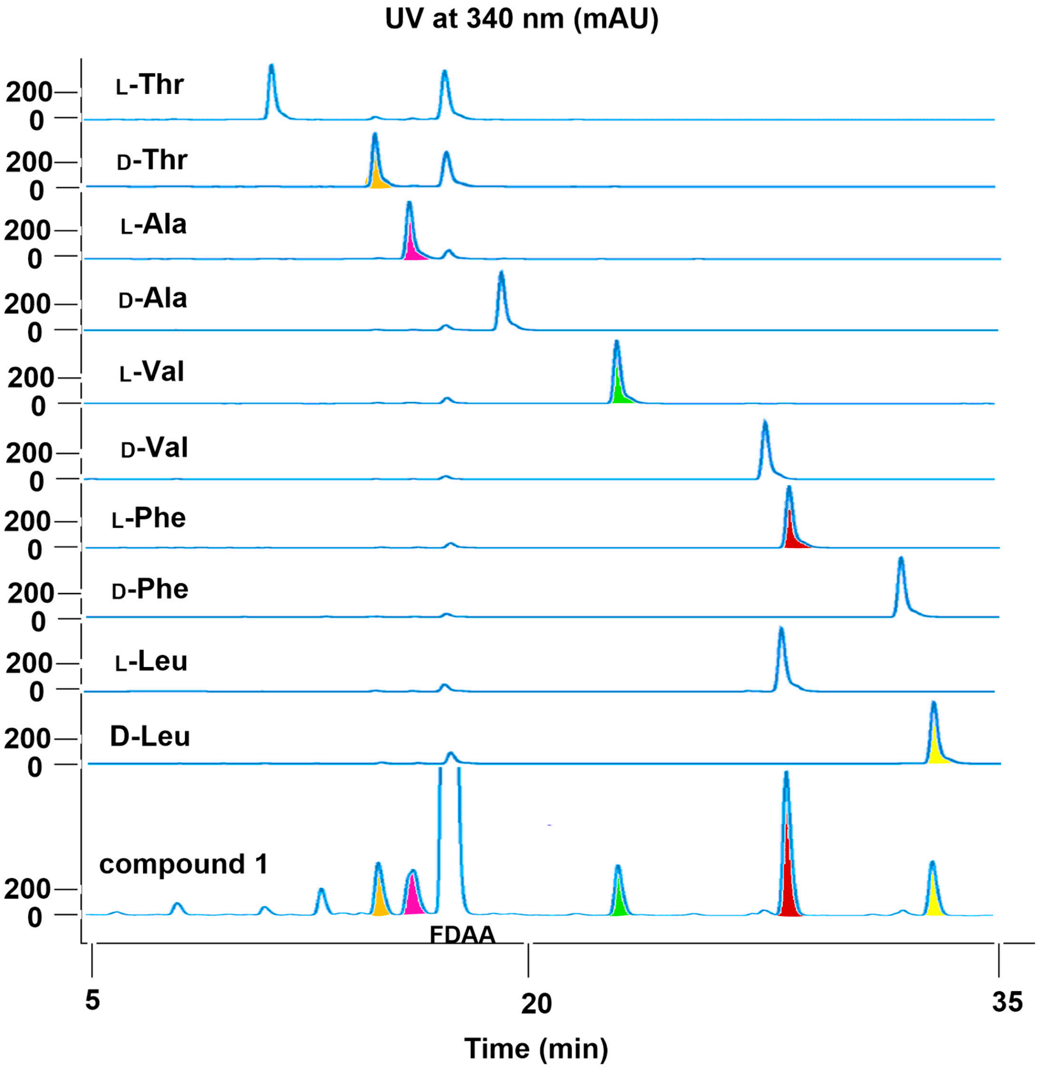

Figure S30. HPLC profile of FDAA-derivatives of nobilamide R3 (**2**) and standard amino acid.

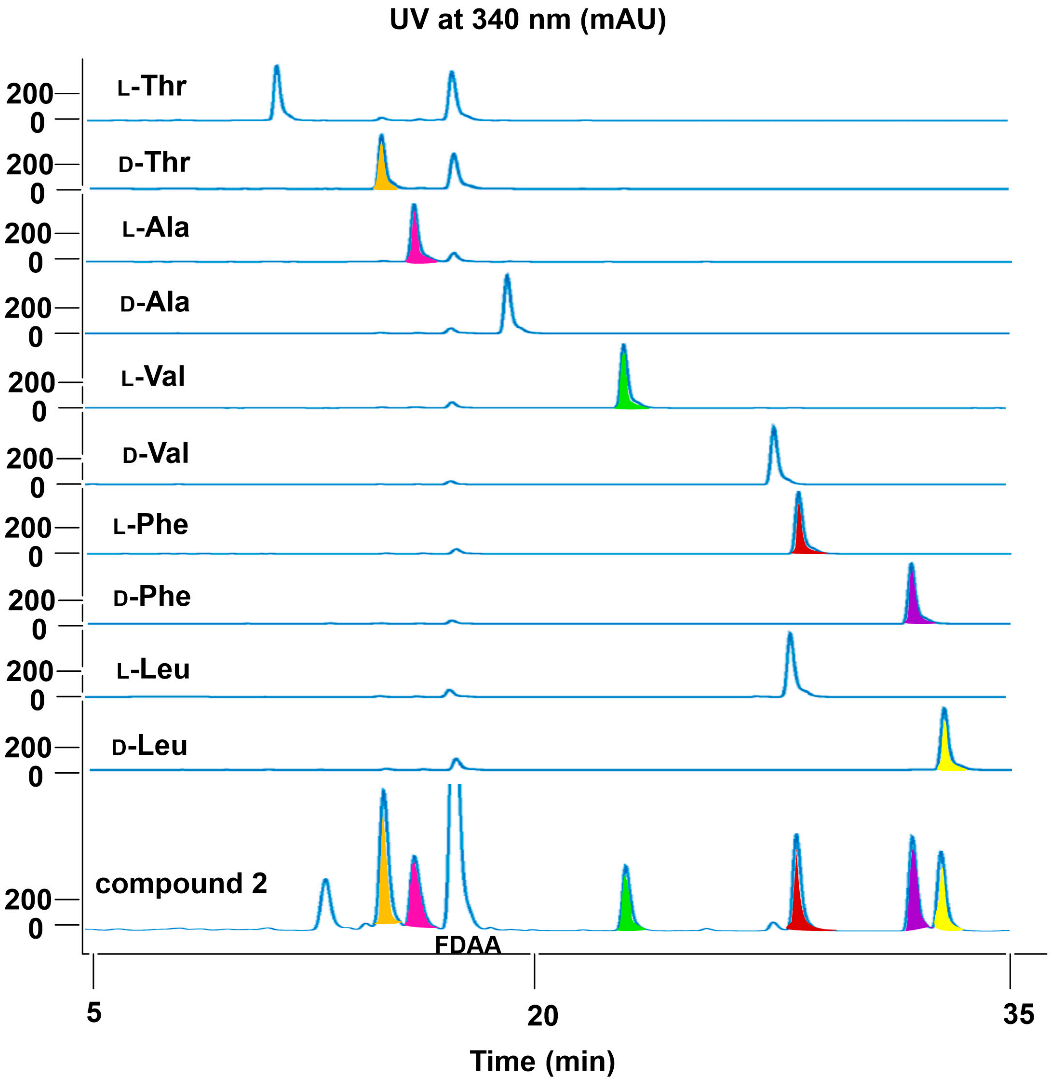

Figure S31. HPLC profile of FDAA-derivatives of A-3302-B (**3**) and standard amino acid.

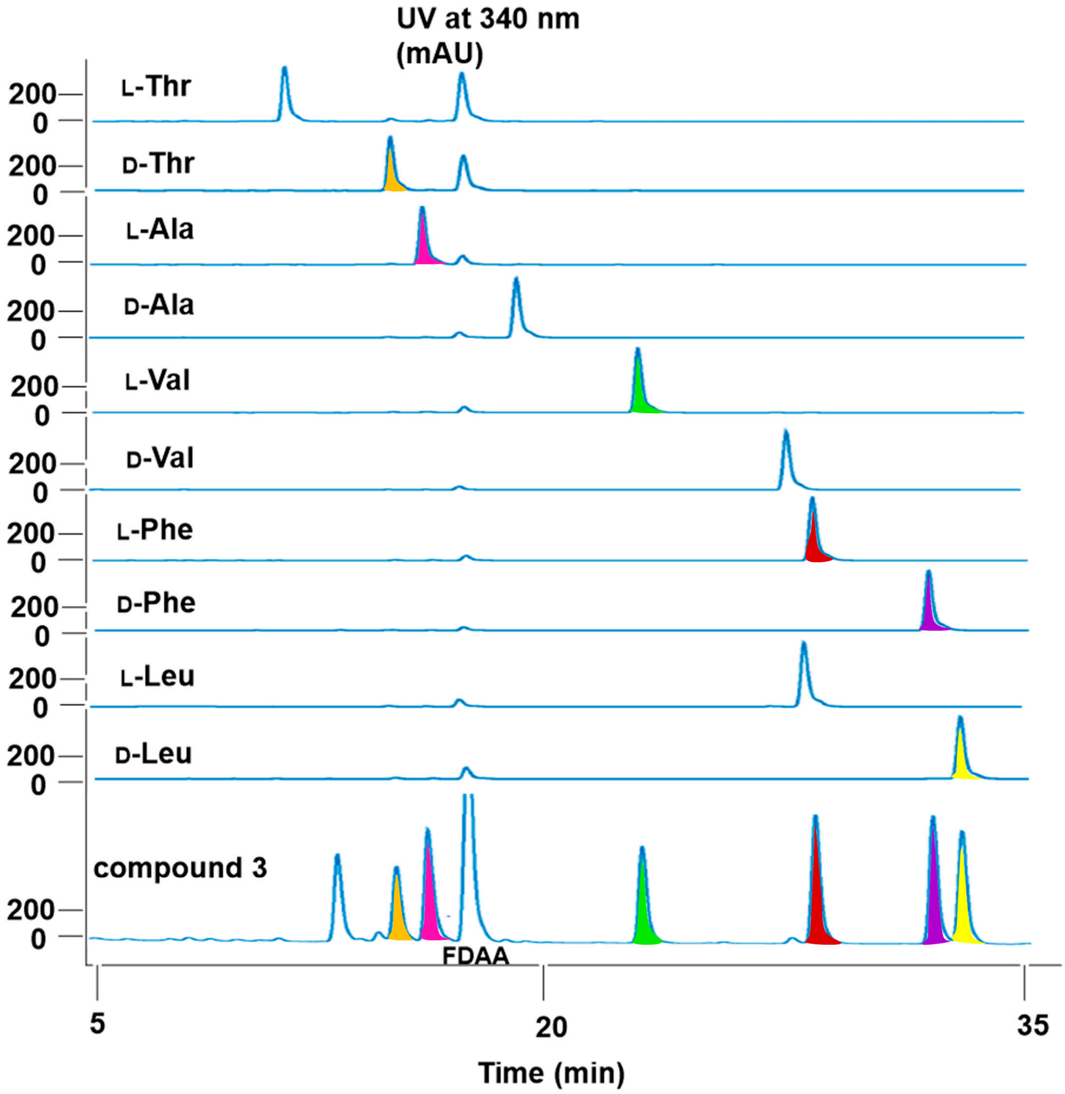

Supplement: Supplementary file 1 [file molecules-31-00547-s001.zip › molecules-4062317-supplementary.pdf]
